# A Programmable KillSwitch in NewTAML Catalysts Decouples Oxidative Reactivity from Catalyst Lifetime

**DOI:** 10.1021/jacs.6c11419

**Published:** 2026-10-07

**Authors:** Debojyoti Chakraborty, Minerva G. Schafer, Xiaowei Ma, Chris Birch, Carlos Felipe Mejía, Rubén A. Toscano, Ronan Le Lagadec, Maxime A. Siegler, Kevin Noonan, Alexander D. Ryabov, Terrence J. Collins

**Affiliations:** † Institute of Green Science (IGS), 6612 Carnegie Mellon University, Pittsburgh, Pennsylvania 15213, United States; ‡ Carnegie Mellon University, Pittsburgh, Pennsylvania 15213, United States; § Instituto de Química, Universidad Nacional Autónoma de México, Circuito Exterior s/n, Ciudad Universitaria, Ciudad de México 04510, Mexico; ∥ Department of Chemistry, The Johns Hopkins University, Baltimore, Maryland 21218, United States

## Abstract

Bis-amido-bis-sulfonamido, [Fe­{4-NO_2_C_6_H_3_-1,2-(NCOCMe_2_NSO_2_)_2_CR_1_R_2_}­(OH_2_)]^−^ [**2**; R_1_/R_2_ = H/H (**2a**), H/CH_3_ (**2b**), CH_3_/CH_3_ (**2c**)], are NewTAML (NT) iron­(III) oxidation catalysts. Herein we identify and mechanistically characterize a ligand-centered, catalyst-lifetime-controlling site (CR_1_R_2_), termed a KillSwitch (KS), that enables balancing technical performance with potential safety constraints in NT/Oxidant process design. Under catalytic conditions, CR_1_R_2_ sites containing two or one hydrogen(s), (**2a**, **2b**, respectively), undergo deprotonation to form electron-rich ylides that accelerate NT oxidative degradation. The **2a**–**c** series defines a KS susceptibility gradient arising from differential C–H bond acidities controlling the ylide-forming deprotonation—**2a**, **2b**, and **2c** represent high-susceptibility-, intermediate-susceptibility-, and null-susceptibility KS states, respectively, thereby providing substantial catalyst functional lifetime control with **2a** < **2b** ≪ **2c**. Kinetic analysis gives similar **2a**–**c** rate constants for H_2_O_2_ activation (*k*_I_) and substrate oxidation (*k*_II_), while catalyst inactivation rate constants (*k*_i_) increase markedly and differentially with increasing pH for **2a** and **2b**, but remain pH-independent for **2c**, which degrades more slowly through a separate pathway. Access to null-KS **2c**—essential for testing the KS hypothesis—required a decade-long synthetic effort culminating in the quantitative methylation of deprotonated **2b**. The KS is expected to be transferable to other NTs with oxidative reactivities distinct from **2a**–**c**, enabling a new dimension in catalyst design that broadly decouples catalytic oxidative aggression from catalyst lifetime. The KS thus provides a practical, safe, and sustainable by design strategy for balancing technical performances against catalyst lifetimes, should shorter-lived NTs become desirable in response to discoveries of postrelease NT adverse environmental effects.

## Graphical abstract

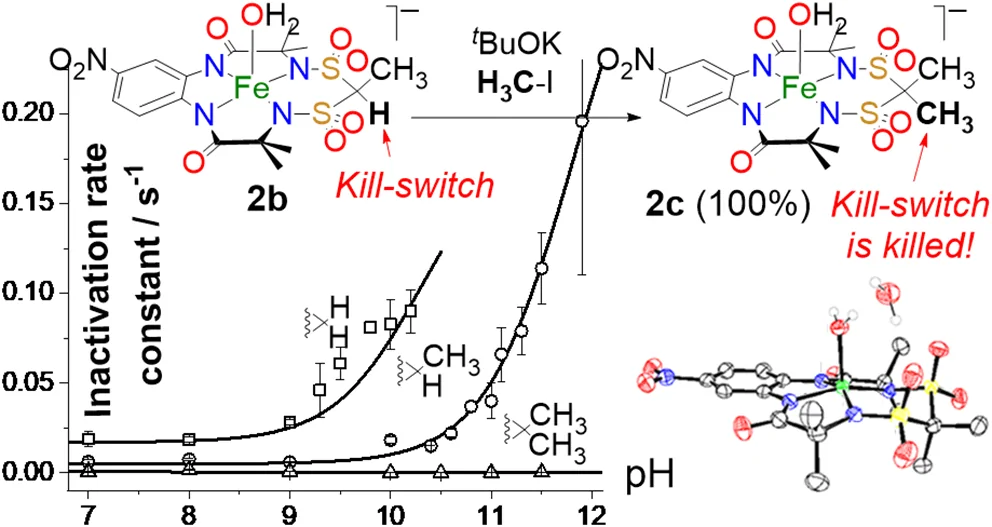

## Introduction

Iron-TAML activators are unique among synthetic small-molecule transition metal catalysts operating effectively in water under ambient conditions at ultradilute catalyst (low nM) and very low oxidant (e.g., H_2_O_2_, OCl^–^, 30 μM)^1^ concentrations while mimicking and outperforming the catalytic cycle of peroxidase enzymes. A key transition in TAML design occurred in 2013 with replacement of the tetra-amido ligand framework of **1** by the bis-amido-bis-sulfonamido motif of **2a**–**c** (R_1_, R_2_ = H or Me; Chart 1).^2^ These NewTAMLs (NTs) are early members of a vast group of patented compositions^3^ that were designed following the realization that the functional lifetimes of earlier TAMLs are likely controlled not by oxidative decay, as long assumed, but by amido-*N* ligand perhydrolysis. Blocking this pathway by replacing what we deduced would be the perhydrolytically most sensitive amide CO groups in the flexible six-membered “tail” ring with an insensitive analogue (SO_2_ initially as herein)^2,4^ was expected to extend NT catalyst lifetimes beyond those of classical TAMLs (“OldTAMLs”).

**Chart 1:**
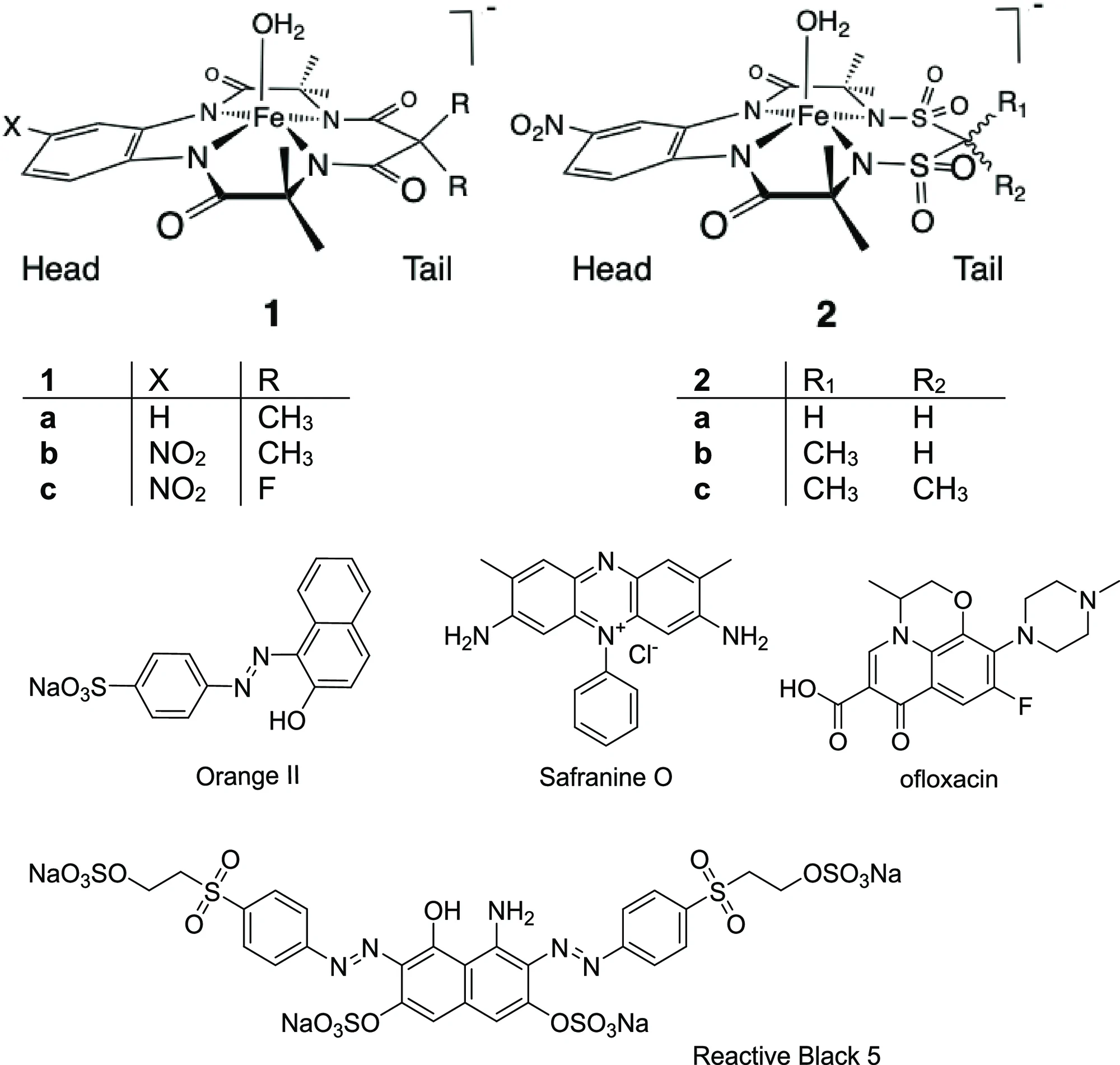
OldTAML Tetra-amido (**1**), NewTAML (NT) Bis-amido-bis-sulfonamido (**2**) Activators of Peroxides, and Organic Dyes Used Or Discussed Herein

Initial results, however, were disappointing. The first produced NT, **2a**, exhibited improved initial rates for H_2_O_2_ activation and dye bleaching (Orange II) and shifted optimal activity toward neutral pH—both desirable for environmental applications. Yet **2a** showed a shorter functional lifetime than the benchmark TAML **1c** (R = F), previously considered the best-in-class performer.^5^ However, commercial development of catalyst **1c** was rejected by us predicated on precautionary concerns regarding its fluorinated structure.^6,7^ Today, **1c** meets the Organization for Economic Co-operation and Development (OECD) definition of Per- and polyfluoroalkyl substances (PFAS).^8^ By suggesting that blocking amido-*N* perhydrolysis on the tail ring had not blocked the lifetime-limiting decomposition pathway, this result left the NT design program without clear direction for performance improvement. The hypothesis that the unchanged two amido-*N* headgroup ligands were sites of initial perhydrolysis was considered unlikely because of the increased ring-strain demands of quaternizing amide carbonyl carbons in five- versus six-membered rings.

This discrepancy between expectation and reality prompted a rethinking of potential degradation pathways. We realized that the C–H sites of the newly introduced six-membered bis-sulfonamido-*N* tail ring might be sufficiently acidic to deprotonate under catalytic conditions—particularly in the most electronegative states of iron, i.e., the high-valent iron-oxo Activated Catalyst states—forming electron-rich ylides that would be susceptible to faster oxidative degradation giving a new degradation pathway that would camouflage an achieved suppression of amido-*N* perhydrolysis. This thinking was quickly supported by the observation of facile C–H/D exchange^2^ at the −SO_2_–CH_2_–SO_2_– position in the Fe­(III) resting state of **2a** to open the concept of a ligand-centered, programmable KS, whereby systematic modulation of C–H acidity in a series of TAMLs could be developed to control NT lifetime almost independently of NT oxidative activity.

To test this concept, we targeted the series **2a**–**c** [R_1_/R_2_ = H/H (**2a**, high-susceptibility-KS), H/CH_3_ (**2b**, intermediate-susceptibility-KS), CH_3_/CH_3_ (**2c**, Null-KS)], predicting a pH dependent monotonic decrease in relative inactivation rate constants *k*_i_ with **2a** > **2b** ≫ **2c**. Catalyst **2b,** with its less acidic than **2a** C-H, was readily prepared and exhibited extended lifetime relative to **2a**, with monotonically increasing differences as the pH was increased, consistent with the proposed deprotonation-driven decomposition mechanism.

Definitive evaluation of the KS required access to the fully deactivated Null-KS variant **2c**. Direct synthesis proved challenging due to the absence and encountered difficulty of preparing suitable precursors such as (ClSO_2_)_2_CMe_2_. Ultimately, **2c** was obtained via the novel synthetic protocol of methylating deprotonated **2b** in its ferric resting state, a solution that came at the end of a decade of on-again/off-again dead-end synthetic efforts.

Here we report (i) quantitative conversion of **2b** to **2c** using ^*t*^BuOK/methyl iodide; (ii) isolation and full characterization of **2c**, including by single crystal X-ray crystallography; (iii) kinetic analysis demonstrating the predicted trend in *k*_i_ (**2a** > **2b** ≫ **2c**) while preserving similar H_2_O_2_ activation (*k*_I_) and substrate oxidation (*k*_II_) rates across the series; (iv) pH-dependent inactivation for **2a** and **2b**, absent in **2c**, which instead undergoes slower degradation via an alternative yet to be identified pathway, *to fully validate the design hypothesis that delivered NTs*; (v) mechanistic studies of the dynamics of KS inactivation; (vi) mechanistic studies of the dynamics and relative technical performance kinetics across multiple important substrates, including test dyes (Orange II and Safranine O) and commercially and sustainability significant compounds such as the fluoroquinolone antibiotic and micropollutant, ofloxacin, and the widely used synthetic dye Reactive Black 5.

These results establish the KS as a ligand-centered, mechanistically defined control element that enables independent tuning of catalyst lifetimes, both by NT choice in the **2a**–**c** series and by pH control within the applied chemistry of each catalyst.

## Experimental Section

### Materials

Orange II (certified pure), pyridine (99%), and KOH (99.98%) were purchased from Acros Organics and used as received. Methyl *p*-toluenesulfonate (MeOTs, 98%) was purchased from TCI. ^*t*^BuOK, methyl iodide (99%, stabilized with Cu), and [PPh_4_]Cl (98%) were Thermo Fisher Scientific reagents. Reactive Black 5, triethylamine, and trimethyloxonium tetrafluoroborate ([Me_3_O]­BF_4_) were purchased from Sigma-Aldrich and used as received. High-purity grade silica for flash chromatography (pore size 60 Å, 220–440 mesh particle size and 35–75 μM particle size) was purchased from Sigma-Aldrich. Safranine O (highly pure biological strain), ethyl acetate (ACS grade), methanol (ACS grade), water (HPLC-grade), acetonitrile (HPLC-grade), and tetrahydrofuran (ACS grade solvent) were obtained from Fisher Chemicals. K_2_HPO_4_, NaHCO_3_, LC-MS grade water, MeOH, KH_2_PO_4_, and Na_2_CO_3_ were purchased from VWR Chemicals. Hydrogen peroxide (30%, for analysis) was purchased from Supelco. NewTAMLs **2a** and **2b** were gifted generously by Sudoc, LLC.

Stock solutions of Orange II and Safranine O (both 6.9 × 10^–4^ M) were prepared by dissolving solid dyes in HPLC grade water, the measured extinction coefficients (ε) being 2.0 × 10^4^ M^–1^ s^–1^ (485 nm, pH 7.0, 0.01 M phosphate) and 2.1 × 10^4^ M^–1^ s^–1^ (519 nm, pH 7.0, 0.01 M phosphate), respectively. Stock solutions of **2a**–**c** (ca. 5.76 × 10^–6^ M) were prepared by dissolving the samples in HPLC grade water; the stock solutions were stored under refrigeration. Stock solutions of H_2_O_2_ (0.3–0.4 M) were also made in HPLC grade water. The concentrations were standardized every 1–2 days by UV–vis spectroscopy at 230 nm using ε = 72.8 M^–1^ s^–1^.^9^ All stock solutions were stored under refrigeration at 4 °C.

### Instrumentation

All pH measurements were performed using a Fisher Scientific Accumet AB-15 pH meter. UV–vis spectra were recorded using an Agilent Cary 3500 Multicell Peltier spectrophotometer. Chromatographic analyses of ofloxacin were carried out on a Shimadzu HPLC system with Shimadzu SCL-40 system controller, LC-40D XS pump, DGU-405 degasser, SPD-M40 diode array detector, CTO-40C column oven, and SIL-40C XS autosampler and a Phenomenex Kinetex EVO C18 100 Å (100 × 4.6 mm, 5 μm) column. The mobile phase consisted of phosphate buffer (0.01 M, pH 3.0) and acetonitrile (92:8, v/v) delivered at a flow rate of 1.0 mL min^–1^. The column oven temperature was maintained at 40 °C; detection was performed at 287 nm. This method was adapted from a previously reported procedure.^1,10^

### Synthesis of **2c**

NewTAML **2b** (50 mg, 0.09 mmol) was dissolved in dry THF (5 mL) in a capped glass vial and mixed with ^*t*^BuOK (205 mg, 1.83 mmol) also dissolved in dry THF (5 mL). The mixture was stirred for 1 h at room temperature and then overnight after addition of methyl iodide (0.1 mL, 45 mmol). All the above operations were performed under inert atmosphere (N_2_) in a glovebox. The reaction progress was followed by ESI-MS by monitoring progress from the reacting **2b** anion (*m*/*z* 529.00) and forming product **2c** (*m*/*z* 543.19). Once complete, stirring was stopped to allow the precipitated KI to separate. The KI was removed by filtration through Whatman 1 filter paper. Cold THF was used to rinse the KI residue. THF and other volatile compounds were then evaporated using a rotatory evaporator. The resulting brown residue was dissolved in ethyl acetate (5 mL) and transferred to a 2 cm (diameter) x 6 cm (height) column packed with silica. The column was sequentially rinsed with hexane, hexane/EtOAc (1:1) and EtOAc to remove impurities. Then product **2c** was eluted with a 70% EtOH/30% EtOAc mixture. The orange band was collected and the solvent was evaporated to dryness. The sticky dark brown material was triturated with diethyl ether to obtain crude **2c** as a dark brown powder. This was dissolved in EtOAc (20 mL), and hexane was added dropwise at 55 °C. After the solution turned opaque it was stored at −20 °C for 48 h. The resulting dark brown solid precipitate was filtered, washed with cold EtOAc, and dried under vacuum for 24 h to obtain solid [Na]­[**2c**] which is mentioned as **2c** elsewhere. Yield: 40 mg, 79%. UV–vis (methanol) λ_max_ = 336 nm (ε = 12,600 M^–1^ cm^–1^); (water) λ_max_ = 356 nm (ε = 8900 M^–1^ cm^–1^); *m*/*z* (negative ion mode) calculated: 543.018, found: 543.018; Anal. Calcd for C_20_H_31_FeN_5_O_9_S_2_Na · EtOAc: C, 37.51; H, 4.65; N, 10.41; found: 37.63; H, 4.84; N, 10.71%.

### Single Crystal X-ray Crystallography

**2c** (20 mg, 0.036 mmol) was dissolved in 1.5 mL HPLC grade water and [PPh_4_]Cl (16.2 mg, 0.043 mmol, 1.2 equiv) was added. The resulting dark orange precipitate was filtered, dissolved in a minimal amount of hot ethanol/water (1:1) mixture and allowed to stand overnight in a small, uncapped vial at room temperature to obtain X-ray quality crystals of [PPh_4_]­[**2c**].

All reflection intensities for [PPh_4_]­[**2c**]_EtOH_ were measured at 110.00 K using a Rigaku XtaLAB Synergy R (equipped with a rotating-anode X-ray source and HyPix-6000HE detector) with CuKα radiation (λ = 1.54178 Å) under the program CrysAlisPro (Version CrysAlisPro 1.171.42.49, Rigaku OD, 2022). The same program was used to refine the cell dimensions and for data reduction. The structure was solved with the program SHELXT-2018/2 and was refined on *F* ^2^ with SHELXL-2019/3.^11^ Analytical numeric absorption correction based on a multifaceted crystal model was applied using CrysAlisPro.^12^ The temperature of the data collection was controlled using the Cryostream 1000 from Oxford Cryosystems. The H atoms were placed at calculated positions (unless otherwise specified) using the instructions AFIX 43 or AFIX 137 with isotropic displacement parameters having values 1.2 or 1.5 *U*_eq_ of the attached C atoms. The H atoms attached to OnW (*n* = 1–4) were found from difference Fourier maps, and their coordinates were refined pseudo freely using the DFIX instructions to keep the O–H and H···H distances within acceptable ranges. The asymmetric unit contains three lattice water solvent molecules. One site contains some amount of very disordered lattice solvent molecules (or mixture of solvent molecules). The contribution of that site was removed from the final refinement using the SQUEEZE procedure in Platon.^13^

Dark orange crystals of [PPh_4_]­[**2c**]_MeCN_ suitable for X-ray diffraction crystallography were obtained by slow evaporation of a water/acetonitrile solution. Crystals were mounted on glass fibers. Data were collected at 230 K on a Bruker Apex-II CCD diffractometer using monochromatic graphite Mo Kα (0.71073 Å) radiation. The data integration for [PPh_4_]­[**2c**]_MeCN_ was performed using a triclinic unit cell to yield a total of 119067 reflections, of which 14173 [R­(int) = 0.029] were independent. The frames were integrated with the Bruker SAINT software package.^14,15^ The structure was solved using Patterson’s method, and refinement (full-matrix least-squares) was performed using the SHELXL-2014/7 program.^11^ The CIF files have been deposited in the Cambridge Structural Database under CCDC2558963­([PPh_4_]­[**2c**]_EtOH_) and CCDC2541503­([PPh_4_]­[**2c**]_MeCN_). Copies of the data can be obtained free of charge from www.ccdc.cam.ac.uk.

### Kinetic Measurements

Initial rates of **2**/H_2_O_2_ bleaching of Orange II and Safranine O were measured spectrophotometrically by following decreasing absorbance at 485 and 519 nm, respectively. Data points were obtained at 1 s intervals. Each experiment was allowed to run for at least 10 min. The measurements were mostly performed at [**2**] (0.1–1.0) × 10^–7^ M, [H_2_O_2_] (0.25–5.0) × 10^–3^ M, [dye] (0.25–6.0) × 10^–5^ M in the buffered solutions (0.01 M phosphate or carbonate) at 25 °C. The initial rates were calculated as slopes of linear absorbance versus time plots using the ε values given above. The rate constants *k*_I_ and *k*_II_ were calculated from the data in 3D plots of initial rates versus [H_2_O_2_] and [Orange II] as described previously.^16^ Experiments with Safranine O and data analyses were performed similarly. All initial rates were measured in duplicate. The inactivation rate constants *k*_i_ were obtained from absorbance versus time plots under the regime of incomplete bleaching of Orange II and Safranine O due to inactivation of catalysts **2** under the operating conditions. The details of the calculating routines used are described elsewhere.^17^ The experimental conditions are specified in Table S6 of the Supporting Information (SI). In some cases, and particularly at higher pH, corrections for the noncatalytic bleaching of Orange II and Safranine O were applied by subtracting such from the catalytic bleaching as previously described.^18^ This increased accuracy in determining the rate constants *k*_i_.

The degradation of Reactive Black 5 was studied by following decreasing absorbance at λ_max_ = 597 nm (ε = 23,000 M^–1^ cm^–1^ both at pH 7.0 and 11.0). In 3 mL glass cuvettes, stock solutions of the dye and **2** were combined in pH 7.0, 0.01 M phosphate buffer to achieve final concentrations of dyes and **2** of 2. 0 × 10^–5^ and 1.0 × 10^–7^ M, respectively. The reactions were initiated by adding H_2_O_2_ (2.0 × 10^–3^ M) and were followed for 3 h. In cases when the bleaching stopped, a second aliquot of **2** was added to confirm that catalyst inactivation was responsible for the cessation in bleaching. All Reactive Black 5 degradation experiments were performed in triplicate. The kinetics of ofloxacin degradation by H_2_O_2_ catalyzed by **2a**–**c** were measured by HPLC as previously described in detail.^1^

## Results and Discussion

### Synthesis of NewTAML **2c**

The KS of **2b** was eliminated by reacting it with methyl iodide in THF in the presence of ^*t*^BuOK (eqs 1 and 2).

1 $$2b+{\mathrm{Base}}^{-}⇄{\left[2b‐H\right]}^{n-}+\mathrm{BaseH}$$

2 $${\left[2b‐H\right]}^{n-}+Q‐{\mathrm{CH}}_{3}\to 2c+Q^{-}$$

3 2b+BuOKt+MeI→2c+BuOHt+KI

The apparent simplicity of reaction 3 is somewhat deceiving because **2c** was achieved in quantitative yield only after a tedious search through multiple reagents, reactant ratios and reaction conditions. The optimized process engages methyl iodide as the methylating agent and ^*t*^BuOK as the strong base in THF solvent under argon. The optimal molar ratios of **2b**, MeI and ^*t*^BuOK of 1:500:30 do not appear to be synthetically advantageous primarily because a large amount of KI is generated in the reaction medium. Nevertheless, the workup developed for **2c** affords brownish orange, analytically pure material. Methylating agents other than MeI employed in eq 2, viz. MeOTs and [Me_3_O]­BF_4_, were ineffective. The large excess of ^*t*^BuOK proved to be crucial—practically no **2c** was formed at the 1:2 molar ratio of **2b** and ^*t*^BuOK. Other details regarding the optimization of synthesis of **2c** are presented in Table S1 of the SI. NewTAML activator **2c** was thoroughly characterized by various techniques including single crystal X-ray crystallography.

The synthesis reported here is innovative over all families of TAML activators.^2,19^ A macrocyclic ligand is typically prepared first followed by the insertion of iron into the four-nitrogen cavity. The novelty of the present method lies in using an NT iron­(III) complex as the starting material. Resting NT **2b** underwent no base-promoted catalyst degradation during the synthesis. This suggests that the synthetic strategy developed in this work based on creating a powerful nucleophile can be used for the preparation of other Null-KS NewTAML activators. Two crystal structures (Figure 1) were obtained for [PPh_4_]­[**2c**] by evaporative crystallization from ethanol (EtOH) or acetonitrile (MeCN). There are no noteworthy differences other than the relative disposition of a water solvate molecule in each—comparative details are given in the SI (Figure S1).

**Figure 1:**
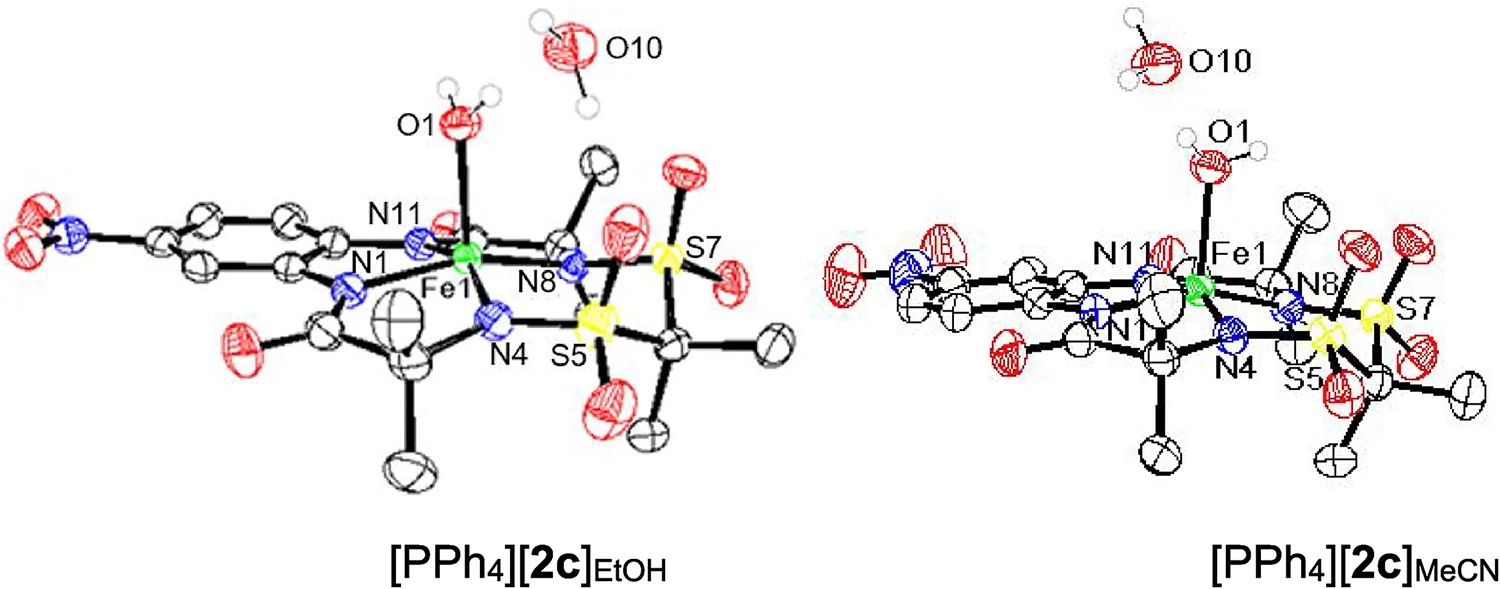
Displacement ellipsoids plots (50% probability level) for [PPh_4_]­[**2c**] crystallized from H_2_O/EtOH, [PPh_4_]­[**2c**]_EtOH_ and H_2_O/MeCN [PPh_4_]­[**2c**]_MeCN_. Hydrogen atoms other than water molecules and the tetraphenyl phosphonium countercation are not shown for clarity.

### Catalysis-Related Properties of **2a**–**c**

Although **2b** is superior, both **2a** and **2b** are excellent oxidation catalysts at circumneutral pH. However, as shown below, a transition from **2a**,**b** to **2c** improves the circumneutral pH technical performance and solves the problem of acquiring truly remarkable NT operational stability at basic pH to fully validate our 2013 responsive insight that a new degradation pathway had to be present in **2a** to explain its higher *k*_i_ value relative to **1c**. Moreover, the finding that **2c** has a longer lifetime than **1c** prompted us to examine the comparative kinetic properties of **2a**–**c** to parametrize the usefulness of the KS in providing a range of lifetimes within the **2** set of NTs.

The measured technical performance parameters (*k*_I_, *k*_II_, *k*_i_) of **2a**–**c** at pH 7 are collected with the catalyst p*K*_a_ values in Table 1. The p*K*_a_s of the aqua complexes and reduction potentials (*E*) for **2a**,**b** have been previously reported^2,20^ and new data for all other columns for **2a**,**b**, and all columns for **2c** complete the comprehensive content of Table 1.

4 $${\left[{\mathrm{LFe}}^{\mathrm{III}}\left(H_{2}O\right)\right]}^{-}⇄{\left[{\mathrm{LFe}}^{\mathrm{III}}\left(\mathrm{OH}\right)\right]}^{2-}+H^{+},\left(K_{a}\right)$$

Since the catalytic activities of NTs correlate with both the p*K*_a_ values of the ferric aqua complexes (eq 4)^6^ and the reduction potentials for the Fe^IV/III^ and Fe^“V”/IV^ transitions in acetonitrile (“V” recognizes ligand noninnocence is more likely to manifest in the most oxidized state in bis­(acetonitrile) than in oxo complexes),^20^ the chosen metrics in Table 1 are especially valuable for rationalizing the comparative catalytic behavior of **2a**–**c** in water at neutral pH with the distinctive *k*_i_ advantage for **2c** at pH 10 highlighted in the final column—the corresponding catalytic behaviors of **2a**–**c** across the pH 6–12.5 range are presented in Figure S3 and Table S2. The *k*_i_ values at pH 10 in Table 1, obtained as described in the following section, alert the reader precisely to the range of control over the pH dependent comparative lifetimes of **2a**–**c** that the KS affords. It is worth noting that the large stabilization of **2c** at high pH has wide ranging commercial value.

**Table 1:** Key Properties of NewTAMLs **2a**–**c** ^a^

New TAML	λ_max_ (ε, M^–1^ cm^–1^) pH 7 water/nm	p*K*_a_ (eq 4)	*E*(Fe^IV/III^)/*E*(Fe^“V”/IV^)/V (Figure S11)^b^	*k*_I_/M^–1^ s^–1^	10^–5^ × *k*_II_/M^–1^ s^–1^	10^3^ × *k*_i_/s^–1^ (pH 7)	10^3^ × *k*_i_/s^–1^ (pH 10)
**2a**	355 (9150 ± 80)	8.8 ± 0.3	1.17/1.59	260 ± 20	3.4 ± 0.7	19 ± 4	83 ± 14
**2b**	355 (10700 ± 200)	8.8 ± 0.1	1.19/1.59	340 ± 10	5.1 ± 0.5	6 ± 1	18 ± 2
**2c**	360 (8600 ± 200)	8.7 ± 0.1	1.20/1.61	410 ± 6	6.9 ± 0.5	5 ± 1	0.10 ± 0.01

a Reduction potentials are in MeCN versus Ag/AgCl. Rate constants *k*_I_ and *k*_II_ (Scheme 1) are at pH 7.0 and 25 °C.

b Measured in MeCN.

At pH 7 (all columns except the last of Table 1), all numerical values for **2a**–**c** are close and do not signal a significant comparative difference regarding the origin of the very diverse pH-dependent *k*_i_ values. In the final column, it is shown that at pH 10.0, *k*_i_ is a factor of 830 if **2a** and **2c** are compared. As will be shown below (e.g., Figure 6), the collective effects of the technical performance parameters (*k*_I_, *k*_II_, *k*_i_) result in **2c** being the distinctly superior technical performer, even at pH 7.

### Catalytic Activity of **2a**–**c**; A Comparative Study

The kinetics of TAML-catalyzed oxidations by peroxides has been extensively studied on multiple occasions. The key steps of the stoichiometric mechanism are presented in Scheme 1.^16,18,21–23^

**Scheme 1:**
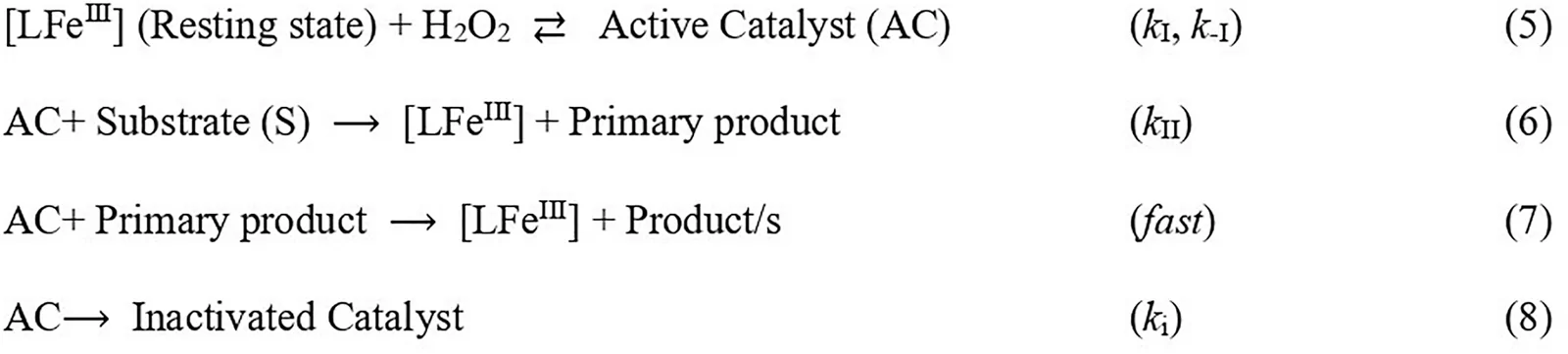
Major Steps of the Stoichiometric Mechanism of NT-Catalyzed Oxidation of Various Substrates by Peroxides in Aqueous Media

The iron­(III)-NT catalyst reacts first with hydrogen peroxide to afford AC (Step 5) which then converts S to a primary product (Step 6). Second order rate constants *k*_I_ and *k*_II_ (*k*_–I_ is assumed to be negligible)^16,24^ are the relevant technical performance parameters within the catalytic cycle and are routinely calculated from typically hyperbolic dependencies of initial rates of substrate (S) depletion as a function of [H_2_O_2_] or [S] (Steps 5–7 predict eq 5; *k*_–I_ ≈ 0). The first-order rate constants *k*_i_ are found by two routines depending on the reaction conditions (see below).^25^

9 $$-\frac{d\left[S\right]}{\mathrm{dt}}=\frac{k_{I}k_{\mathrm{II}}\left[H_{2}O_{2}\right]\left[S\right]}{k_{I}\left[H_{2}O_{2}\right]+k_{\mathrm{II}}\left[S\right]}\left[{\mathrm{Fe}}_{t}^{\mathrm{III}}\right]$$

The rate constants for each catalyst were determined by fitting experimental 3D sets of data of initial rate (−*d*[S]/*dt*) versus [H_2_O_2_] and [S] to eq 5 using Orange II (Chart 1) as the substrate.

Enhanced accuracy and reliability were achieved by using at least 42 data points for each 3D plot (Figure S3 and Table S2). Naturally, prior to a nonlinear least-squares fitting of the experimental data to eq 5, it was confirmed that the initial rates were directly proportional to the concentrations of **2** (Figure S2). Bell-shaped dependencies of *k*_I_ and *k*_II_ on pH are shown in Figure S4.

### A Kinetic Study of the Relative Operational Instabilities of **2a**–**c** That Confirms the Presence of the KS in **2a**,**b**

High values for the rate constants *k*_I_ and *k*_II_ do not automatically imply that an oxidation catalyst will deliver a high technical performance. If the operational instability associated with the rate constant *k*_i_ in Scheme 1 is also high, such that the catalyst undergoes rapid inactivation under the operating conditions, high efficiency within a cycle provides no usable output. Therefore, the rate constants *k*_i_ must also be measured in any kinetics-based assessment of the technical performance of an oxidation catalyst.

The rate constants *k*_i_ are extractable from data as presented in Figure 2. The experiments are set so that there is incomplete dye bleaching resulting from catalyst inactivation. Hence, addition of a new **2c** aliquot resumes the bleaching as in Figure 2A.^17,26^ There are two options for calculating the rate constants *k*_i_. Better accuracy is achieved when the bleaching occurs in the ″*k*_II_ regime″, i.e., when *k*_I_[H_2_O_2_] ≫ *k*_II_[S]. Under these conditions, eq 5 becomes eq 6 and *k*_i_ is calculated by linearization of the entire kinetic trace using eq 7—in bleaching experiments, *S*_t_ and [Fe_t_^III^] are the total concentrations of the dye and the iron­(III) catalyst, respectively; *x* and *x*_∞_ are the concentrations of bleached dye at time *t* and at *t* → ∞, respectively (Figure 2A).^17^

10 $$-\frac{d\left[S\right]}{dt}=k_{\mathrm{II}}\left[S\right]\left[{\mathrm{Fe}}_{t}^{\mathrm{III}}\right]$$

11 $$\ln\left[\ln\left(\frac{S_{t}-x}{S_{t}-x_{\infty }}\right)\right]=\ln\left(\frac{k_{\mathrm{II}}}{k_{i}}\left[{\mathrm{Fe}}_{t}^{\mathrm{III}}\right]\right)-k_{i}t$$

The slope of a linear plot of the left side of eq 7 versus time (Figure 2B) equals −*k*_i_; the intercept allows one to calculate *k*_II_ from a single kinetic run for comparison with the value obtained from the classical steady state treatment explained above.

**Figure 2:**
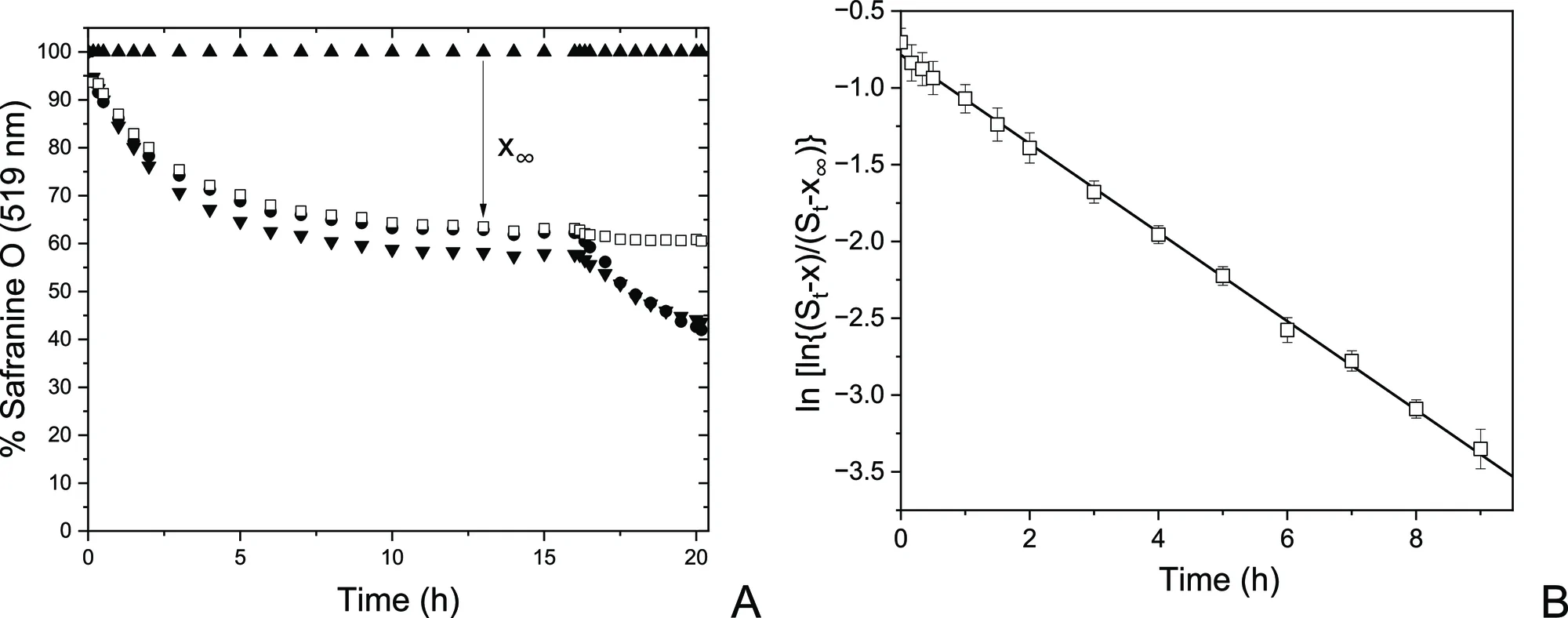
(A) Example of incomplete bleaching of the dye Safranine O (3 × 10^–6^ M) by H_2_O_2_ (5 × 10^–3^ M) in the presence of **2c** (5 × 10^–9^ M) at pH 11.0 and 25 °C. White squares (□) show that addition of another aliquot of H_2_O_2_ (5.0 × 10^–3^ M) does not resume bleaching. Solid circles (●) and inverted solid triangles (▼)­show that the addition of another aliquot of **2c** (5 × 10^–9^ M) resumes the catalytic bleaching. The solid triangles(▲) show the inefficiency of uncatalyzed Safranine O bleaching by H_2_O_2_. (B) Linearization of kinetic data presented in **A** in terms of eq 7.

If the relation *k*_I_[H_2_O_2_] ≫ *k*_II_[S] does not hold and the oxidation occurs either in the ″*k*_I_ regime″ (*k*_I_[H_2_O_2_] ≪ *k*_II_[S]) or in the mixed regime (*k*_I_[H_2_O_2_] ∼ *k*_II_[S]), eq 7 does not hold.^26^ Nevertheless, *k*_i_ can be obtained when an excess of H_2_O_2_ over S is deployed by using eq 8, though with a lower accuracy, because just two data points are used, *viz*. S*_t_* and S_∞_ = S*_t_* – *x*_∞_, and a higher uncertainty is always present in measuring S_∞_.^26^

12 $$\ln\frac{S_{t}}{S_{\infty }}=\frac{k_{\mathrm{II}}}{k_{i}}\left[{\mathrm{Fe}}_{t}^{\mathrm{III}}\right]$$

The *k*_II_ kinetic regime (*k*_I_[H_2_O_2_] ≫ *k*_II_[S]) can be enforced by using higher concentrations of H_2_O_2_ and/or a less reactive S. Higher pH usually favors the *k*_II_ regime as well. Safranine O is advantageous compared to Orange II at higher pH because it is more resistant to the NT-catalyzed oxidation.^17^

It was found for **2b** and **2c** at pH 7.0 that both dyes afford similar values of *k*_i_. Either eq 7 or eq 8 was used depending on the ratio between *k*_I_[H_2_O_2_] and *k*_II_[S]. Thus, the rate constants *k*_i_ at pH 7.0–11.0 and 25–60 °C were determined using eqs 7 or 8 and Orange II or Safranine O. All *k*_i_ values measured at 25 °C are shown cumulatively in Figure 3A, where the symbols and colors indicate the equation and the dye used.

**Figure 3:**
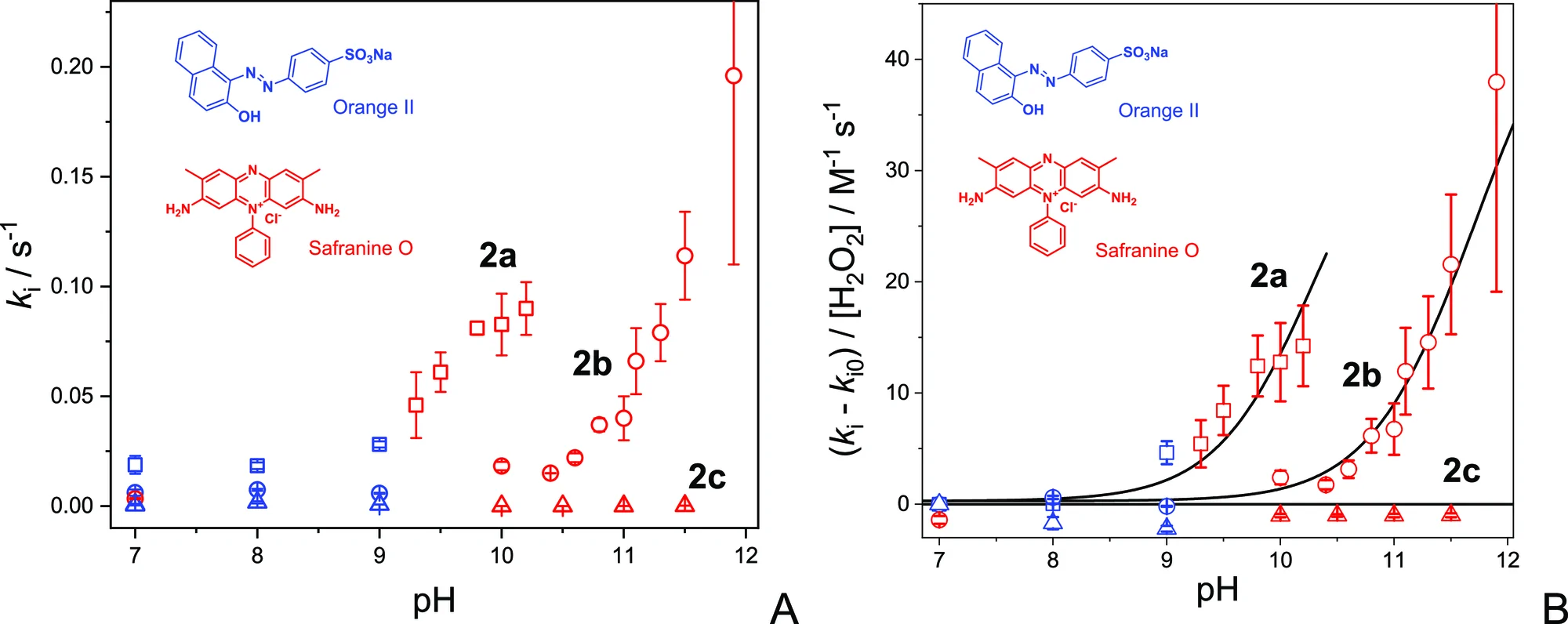
(A) pH Profiles for the rate constants *k*_i_ for **2a** (squares), **2b** (circles) and **2c** (triangles). (B) Same data as in **A** replotted as $\frac{k_{i}-k_{i0}}{\left[H_{2}O_{2}\right]}$ versus pH. The solid lines were calculated using the best-fit values of the rate and equilibrium constants in Table 3. Conditions: 25 °C, 0.01 M phosphate (pH 7–8) or carbonate (pH > 8) buffers. Colors of symbols indicate the dye used (Orange II, eq 8 or Safranine O, eq 7). The values of *k*_i_ were obtained over the range of concentrations of dyes (0.5–3.0) × 10^–6^ M, H_2_O_2_ (1.5–5.0) × 10^–3^ M and **2** (0.01–1) × 10^–7^ M. The exact concentrations used for acquiring the data points are collected in Table S6.

The new catalyst **2c** is resistant to suicidal inactivation under the operating conditions at pH 7, *k*_i_ was very low and could not be measured at 25 °C. Incomplete bleaching of Orange II by **2c**/H_2_O_2_ was observed at 40 °C and above and *k*_i_ values were calculated in the 40–60 °C range using eq 8. The latter requires rate constants *k*_II_ which were calculated from the 3D plots in Figure S5 and collected in Table S3. The values of *k*_i_, found according to eq 7 at pH 7.0 (incomplete bleaching pattern in Figure S6), were extrapolated to 25 °C using the Eyring equation (Figure S7). The calculated value of *k*_i_ for **2c** was used in Figure 3A (details in SI) and the enthalpies and entropies of activation for **2c** are listed in Table 2. The enthalpy of activation Δ*H*‡ for *k*_i_ is higher than that for *k*_II_ which permits observation of the inactivation of **2c** by raising the temperature.

**Table 2:** Activation Parameters Δ*H* ^≠^ (kJ mol^–1^) and Δ*S* ^≠^ (J mol^–1^ K^–1^) Correspond to the Rate Constants *k*_I_, *k*_II_, and *k*_i_ for Catalyst **2c** at pH 7.0

Rate constant	Δ*H* ^‡^	Δ*S* ^‡^
*k*_I_	28 ± 3	–100 ± 9
*k*_II_	17 ± 1	–77 ± 5
*k*_i_	28 ± 4	–195 ± 13

The rate constants *k*_i_ plotted against pH in Figure 3A show that the major goal of the present project was successfully reached. Null-KS-**2c** without hydrogens in its tail part is much more resistant to inactivation under the operating conditions in terms of *k*_i_ compared to high-susceptibility-KS-**2a** and intermediate-susceptibility-KS-**2b**. The *k*_i_ values decrease as anticipated in the series **2a** > **2b** ≫ **2c** over the entire pH range explored. Importantly, *k*_i_ values for Null-KS-**2c** are pH insensitive, at least up to pH 11.5. This feature makes **2c** useful under basic conditions where neither **2a** nor **2b** can be usefully deployed (see below). Several finer additional observations are also noteworthy; (*a*) *k*_i_ for **2c** tends to diminish as pH grows—this is hard to see in Figure 3 and therefore the numerical values with changing pH are listed in Table S4, and (*b*) the rate constant *k*_i_ for **2c** does not depend on the amount of H_2_O_2_ in solution at pH 11.0, whereas that for **2b** does (Figure 4). The observed linear dependence for **2b** with an intercept suggests the expression for *k*_i_ given in eq 9. The operational instability of **2a** at high pH is too high to permit *k*_i_ measurements as a function of [H_2_O_2_]. The calculated rate constants *k*_i0_ and *k*_i,H_2_O_2__ are collected in Table S5 together with the previously reported values for TAMLs **1a** and **1b** (Chart 1).^17^ The baseline first-order inactivation rate constant (*k*_i0_) quantifies intrinsic catalyst degradation when kill-switch-mediated inactivation is negligible (e.g., at circumneutral pH or under very low H_2_O_2_ concentration), thereby limiting its environmental persistence.

13 $$k_{i}=k_{i0}+k_{i,H_{2}O_{2}}\left[H_{2}O_{2}\right]$$

The first-order term in H_2_O_2_ in eq 9 for **2b** is observed under basic conditions—*k*_i,H_2_O_2__ is negligible at pH 8 (Figure S8). Notably, *k*_i,H_2_O_2__ ≈ 0 for **2c** (Table S5), the Null-KS NT. The values of *k*_i_ are practically constant (Figure 3A) and insensitive to [H_2_O_2_] at pH 7 – 9 for all **2** NTs (Figure S8). Equation 9 can therefore be rewritten as eq 10 because the latter is more appropriate for analysis of the influence of pH on the effective second order rate constant *k*_i,H_2_O_2__ which refers to interactions of the deprotonated KS in the AC with H_2_O_2_ and HO_2_^–^ (Scheme 2). The corresponding dependence of *k*_i,H_2_O_2__ versus pH is presented Figure 3B.

14 $$\frac{k_{i}-k_{i0}}{\left[H_{2}O_{2}\right]}=k_{i,H_{2}O_{2}}$$

The *k*_i0_ values at different pHs in Table S4 underline the enhanced operational stability of **2c** at pH 11. Relative values of *k*_i0_ increase in the series **2c** (1) < **1a** (9) < **1b** (15) < **2b** (314), the highest being observed for **2b** as expected for the KS-containing species. Again, the series does not include **2a** for which *k*_i0_ could not be measured at pH 11 though the anticipated *k*_i0_ should be larger than that for **2b**.

**Figure 4:**
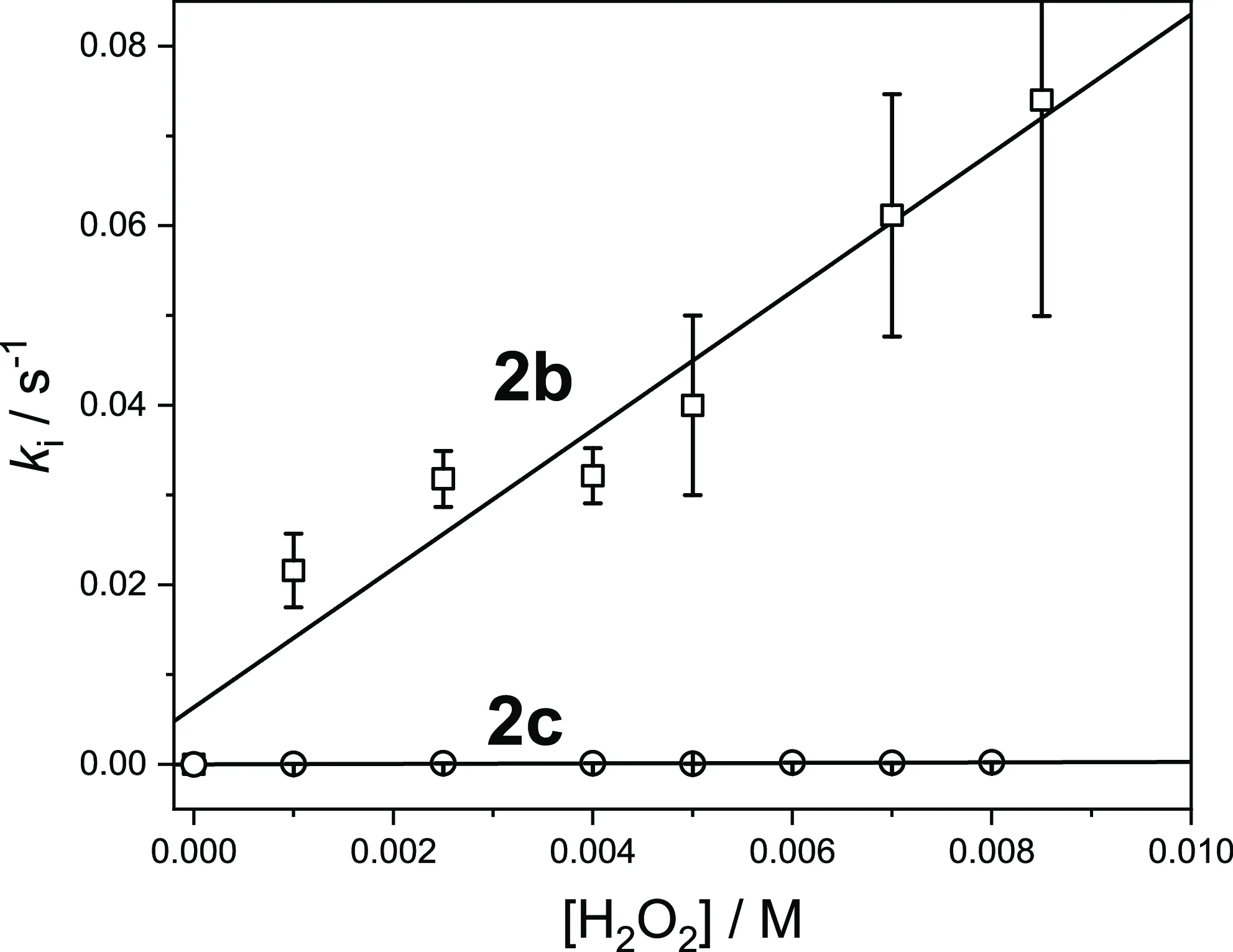
Rate constants *k*_i_ for **2b** and **2c** at variable concentrations of H_2_O_2_ at pH 11.0 (0.01 M carbonate) and 25 °C.

**Scheme 2:**
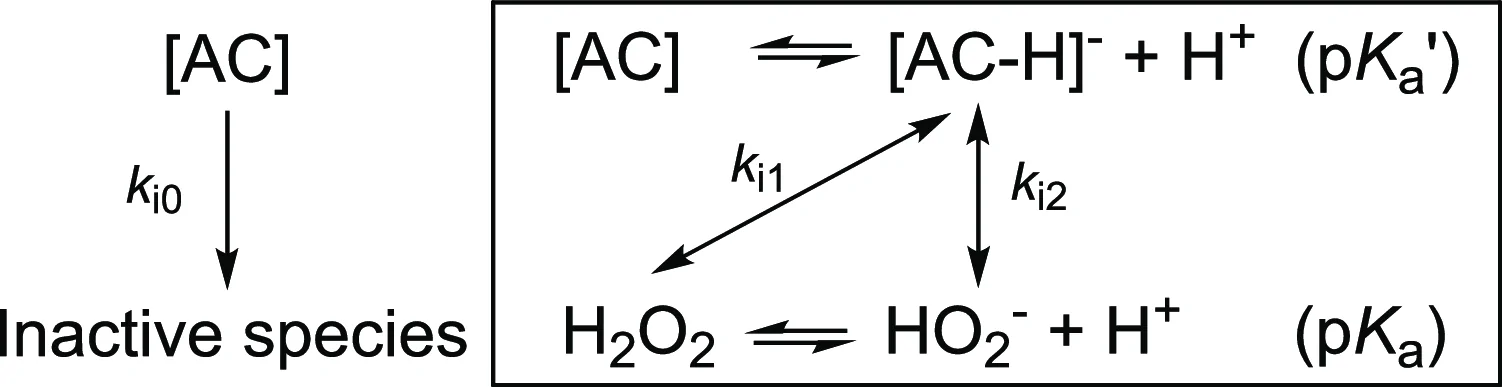
H_2_O_2_-Independent (*k*_i0_) and H_2_O_2_-Dependent (in the Box) pathways (*k*_i1_ and *k*_i2_) of Inactivation of Deprotonated Active TAML Catalysts ([AC-H]^−^) under the Operating Conditions^a^

a Double arrows in the box indicate pairs of interacting species and the corresponding rate constants *k*_i1_ and *k*_i2_ which define interactions leading to inactive species as in the case of *k*_i0_.

Note that peroxide dependent pathways measured by *k*_i,H_2_O_2__ are found for both **1** and **2** catalysts (Table S5). In the case of the **1** generation, all data support that *k*_i,H_2_O_2__ arises from amido-*N* ligand perhydrolysis in the six-membered chelate ring. For **2a** and **2b**, a peroxide-dependent pathway also occurs at the activated KillSwitch, but the case presented here supports instead that at pH 11 suicidal inactivation is faster than **1c** perhydrolysis via the intermediacy of the deprotonated KillSwitch by a mechanism measured by *k*_i0_. In the case of Null-KS **2c**, the inactivation reaction occurs by a yet slower pathway that remains to be determined.

Scheme 2 is key for exploring the effect of acidity on *k*_i,H_2_O_2__ where the boxed component leads to eq 11.

15 $$k_{i,H_{2}O_{2}}=\frac{k_{i}-k_{i0}}{\left[H_{2}O_{2}\right]}=\frac{k_{i1}K_{a}^{\prime }\left[H^{+}\right]+k_{i2}K_{a}K_{a}^{\prime }}{{\left[H^{+}\right]}^{2}+\left(K_{a}^{\prime }+K_{a}\right)\left[H^{+}\right]+K_{a}K_{a}^{\prime }}$$

The data in Figure 3B for both **2a** and **2b** were fit to eq 11 and the fitting results are shown in Figures S9 and S10, respectively. The best-fit numerical values of rate constants (*k*_i1_–*k*_i2_) and equilibrium constants (p*K*_a_ and p*K*_a_’) are collected in Table 3. In the absence of KS activation, interactions between AC/H_2_O_2_ and AC/HO_2_^–^ lack chemical relevance and were therefore excluded from analysis. Simulated solid lines in Figure 3B using the data in Table 3 agree satisfactorily with the experimental results.

**Table 3:** Rate (*k*_i1_–*k*_i2_ in M^–1^ s^–1^) and Equilibrium (p*K*_a_ and p*K*_a_’) Constants eq 11 for **2a** and **2b** at 25 °C in 0.01 M Carbonate Buffer^a^

TAML	10^2^ × *k*_i0_	*k*_i1_	*k*_i2_	p*K*_a_	p*K*_a_’
**2a**	1.9 ± 0.4	31 ± 4	80 ± 6	11.2 ± 0.3	10.2 ± 0.1
**2a**		40 ± 7			10.2 ± 0.1
**2b**	0.6 ± 0.1	15 ± 3	45 ± 4	11.1 ± 0.2	11.7 ± 0.1
**2c**	0.5 ± 0.1				

a Rate constants *k*_i0_ (s^–1^) for **2a**–**c** are included for completeness.

The p*K*_a_ values calculated for **2a** and **2b** agree with each other and are close to the range of 11.2–11.4, typical of H_2_O_2_.^27,28^ The p*K*_a_ of H_2_O_2_ (11.2) is out of the pH range covered (7.0–10.3) for catalyst **2a**, plus *K*_a_’ ≫ *K*_a_. Therefore, fitting the data to eq 11 is possible with *K*_a_ ≈ 0 to simplify eq 11 and the so-derived best-fit values were used to build a theoretical curve for **2a** in Figure 3B.

The absolute p*K*_a_’ values found for KS sites in the ACs seem to be chemically acceptable on the following grounds. The p*K*_a_ of ^*t*^BuOH is 16.5^30^ and the resting state of **2b** has been deprotonated by ^*t*^BuOK (3) in the **2c** synthetic pathway suggesting that the p*K*_a_ of **2b** in the iron­(III) ground state may be around 16–17—note that the synthetic deprotonation/methylation of **2b** is carried out in dry-THF making this connection indirect. AC as it appears in Scheme 1 may be by two oxidation equivalents above the resting state^10,24,29,30^ and the associated increase in electronegativity at iron may decrease p*K*_a_’ of the KS hydrogen by four units as occurs, for example, for just a single oxidation unit state increase in the [Fe­(OH_2_)_6_]^2+^/[Fe­(OH_2_)_6_]^3+^ pair.^31–33^ Thus, p*K*_a_’ = 11.7 found for **2b** comfortably can be ascribed to deprotonation of the KS of the AC (eq 16).
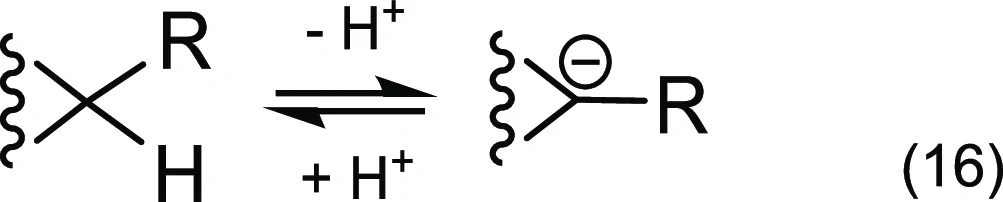


Moreover, it is encouraging that the p*K*_a_’ value of 10.2 for **2a** is by 1.5 units lower than for **2b** as this nicely fits with the increased electron donor capacity of −CH_3_ vs −H. The Δp*K*_a_ = 1.5 for the key structural fragments of **2a** and **2b** matches Δp*K*_a_ for the water/methanol pair, the p*K*_a_ values of which equal 14.0 and 15.5, respectively (Table 4).^31–33^ The 'magic' one and a half difference for chemically diverse molecules with structurally and electronically similar fragments supports the importance of equilibrium 16 for understanding the operational instability of **2a** and **2b** in basic media.

**Table 4:**
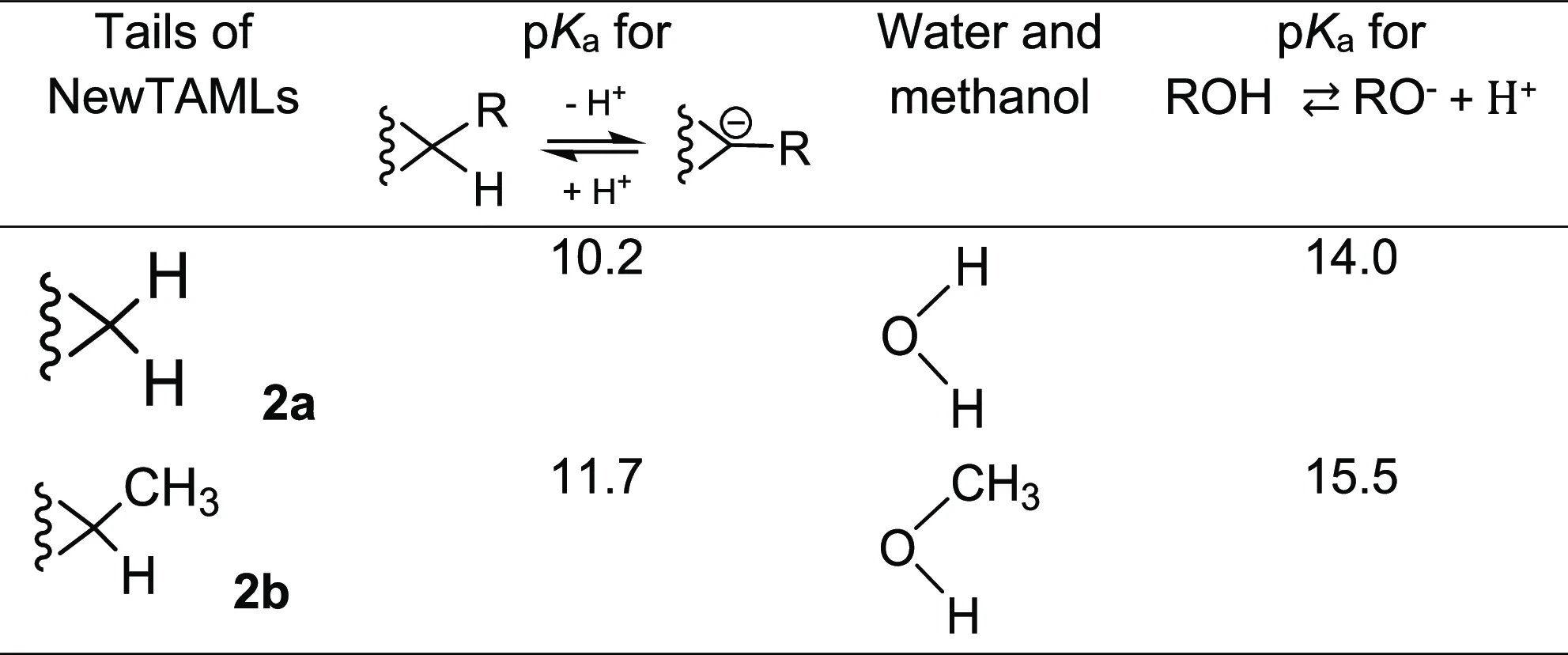
Comparison of the Experimentally Found p*K*_a_ Values of Catalytically Active Forms of NewTAMLs **2a** and **2b** with p*K*_a_’s of Water and Methanol

Kinetic calculation undermines any prediction of the fast steps in catalysis. The kinetic dependencies of *k*_i, H_2_O_2__ on pH and [H_2_O_2_] suggests the deprotonation of the KS and nucleophilic attack by the H_2_O_2_ or HO_2_^–^ are key steps in the deactivation of the catalyst.

### The Superiority in Technical Performance of NewTAML **2c** over **2a** and **2b**

The use of **2c** is that its higher technical performance than **2a**,**b** makes it the clear top choice for every application. Two representative targets for the application of NewTAMLs **2** were selected to illustrate advantages of **2c** versus **2a**,**b**, *viz*. ofloxacin and Reactive Black 5 (RB5) (Chart 1). The former is a widely detected micropollutant eliciting adverse ecological effects even at low concentrations—ofloxacin has appeared on the EU Water Framework Directive Watch List.^34,35^ RB5 is one of the most widely used azo dyes in the textile industry.^36,37^ Substantial releases during textile dyeing lead to highly colored, difficult to treat effluents.^38^ Both are appropriate benchmark compounds for evaluating the technical performances of new oxidation catalysts.

At pH 7.0, the catalytic oxidations with **2b** and **2c** are comparable for the degradation of ofloxacin^10^ (rate of oxidation by **2c** > **2b**) and ofloxacin is easily almost quantitatively oxidized by H_2_O_2_, (Figure 5) catalytically in presence of **2**. Comparable catalytic oxidation can be achieved by **1c**/H_2_O_2_ if at least 10 equiv more **1c** are used with respect to the concentration of **2**. At pH 11.0, the ofloxacin conversion after 3 h reaches only 6 and 15% in the presence of **2a** and **2b**, respectively (Figure 5B). To illustrate that this weak performance arises from the KS, and not from a substrate effect such as inhibition, similar comparisons were made at pH 7.0 with the dye Reactive Black 5 (Figure 6). In this case dye bleaching also remains incomplete after 3 h being 44, 58 and 87% for **2a**–**c**, respectively. Clear advantages of **2c** are achieved at pH 11.0 where the related conversions are 10, 25 and 100% for **2a**–**c**, respectively.

**Figure 5:**
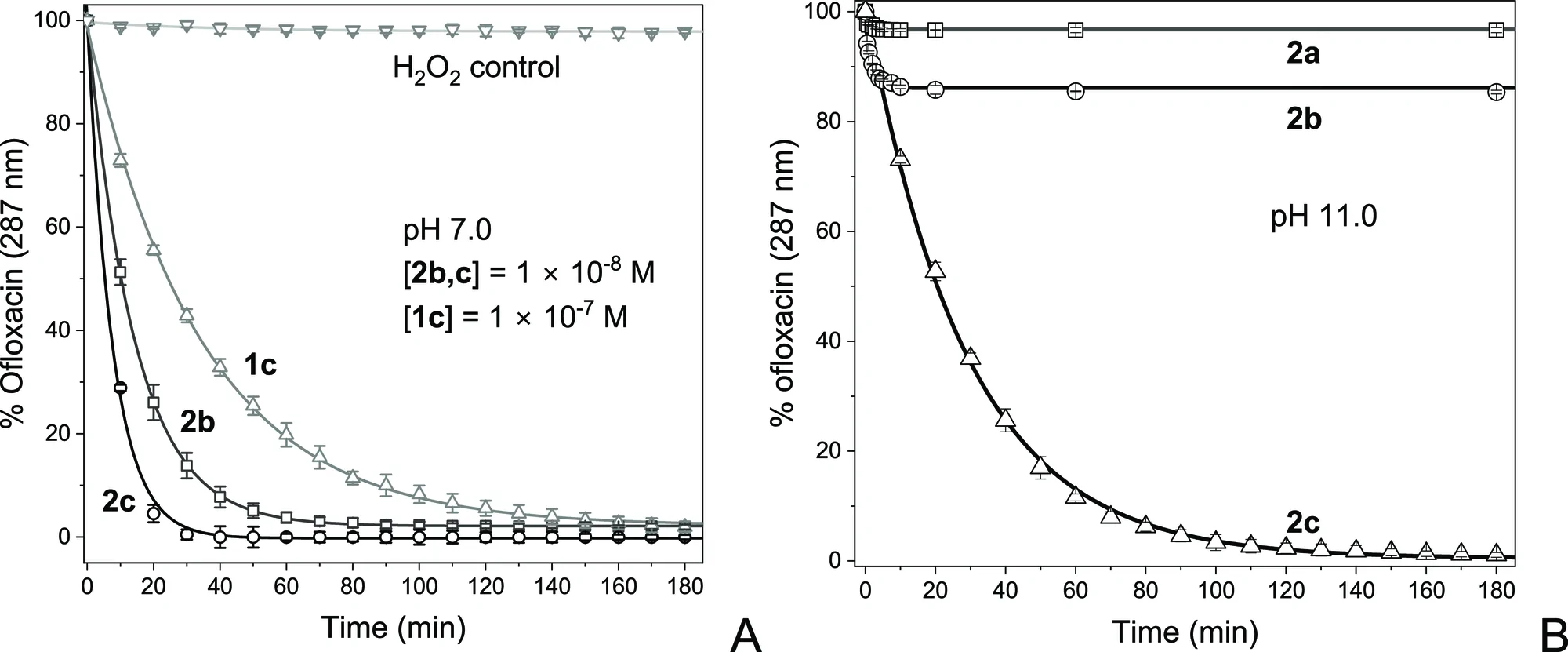
**1c** and **2**-Catalyzed degradation of ofloxacin (2.0 × 10^–5^ M) at pH 7.0 (0.01 M phosphate) (A) and pH 11.0 (0.01 M carbonate) (B). Conditions: [**2**] = 1.0 × 10^–8^ M, [H_2_O_2_] = 1.0 × 10^–3^ M, 25 °C. Data for **1c** and H_2_O_2_ control in (A) adapted from Ma et al.^10^ under CC-BY 4.0 license. Copyright 2025 the Authors. Published by American Chemical Society. Conditions: [ofloxacin] = 3.0 × 10^–5^ M, [H_2_O_2_] = 1.0 × 10^–3^ M, [**1c**] = 1.0 × 10^–8^ M, 25 °C.

Thus, a substantially increased practicality of Null-KS-**2c** has been achieved under basic conditions. The initial rates for **2a**–**c** are close (Figures 5, 6), but **2c** remains intact much longer providing the highest turnover numbers (TONs) for both ofloxacin and Reactive Black 5.

**Figure 6:**
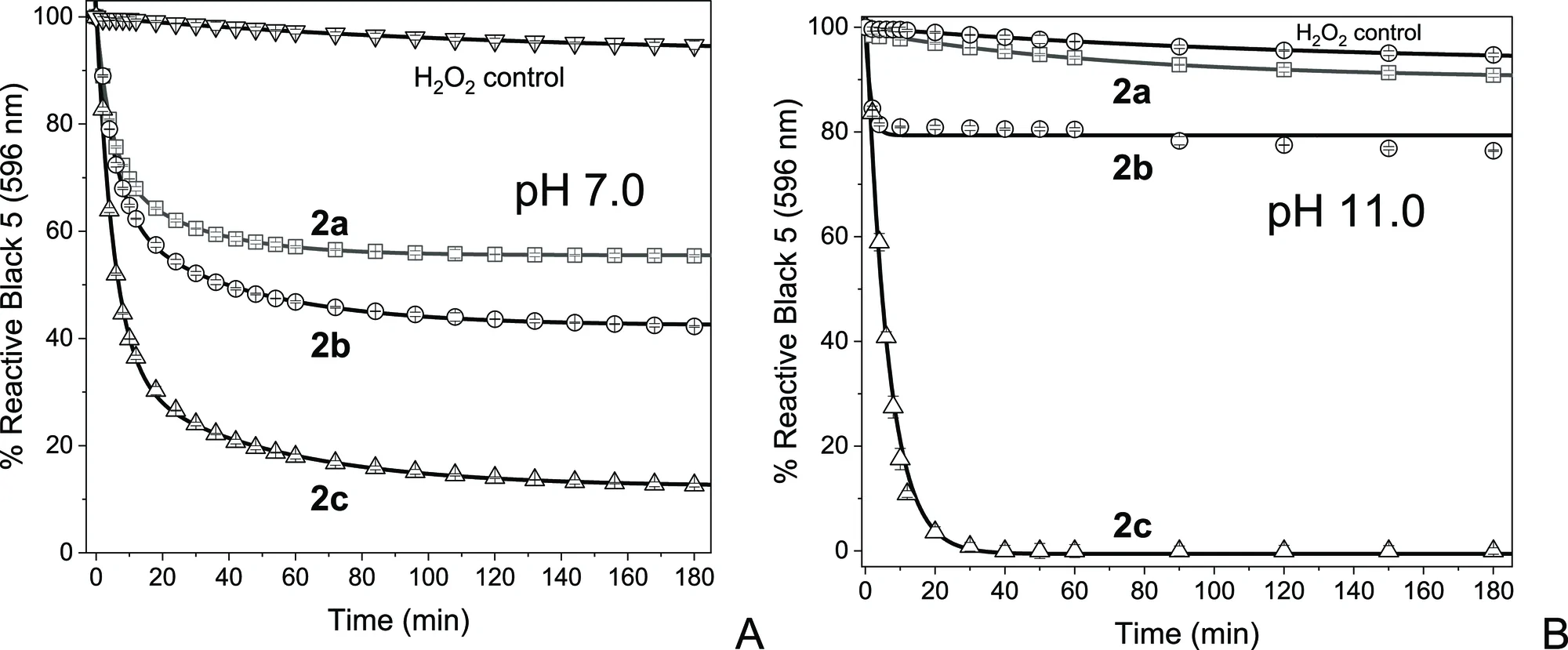
**2**-Catalyzed degradation of Reactive Black 5 (2.0 × 10^–5^ M) at pH 7.0 (0.01 M phosphate) (A) and pH 11.0 (0.01 M carbonate) (B). Conditions: [**2**] = 1.0 × 10^–8^ M, [H_2_O_2_] = 2.0 × 10^–3^ M, 25 °C.

Turnover Numbers (TONs) can be calculated from data such as in Figures 5 and 6 accepting that one is measuring just the reactivity that is focused on the first step in the push toward minerals that NT/H_2_O_2_ typically sets in motion. TONs for TAMLs may also be estimated theoretically for cases with incomplete bleaching provided the rate constants *k*_II_ and *k*_i_ are known because *x*_∞_ = S*_t_*–S_∞_ can be calculated from eq 7 and TON = *x*_∞_/[Fe^III^]*_t_*.^1,39,40^ Measured and calculated TONs for different dyes, NTs and pH are compared in Table 5 and match reasonably closely for most of the cases. Thus, the TONs provide an extra indication of the legitimacy of our general quantitative kinetic model for iron­(III) TAML activators of peroxides operating with three principal determinants of the oxidative catalysis, *i.e*., the in-cycle catalytic activity (*k*_I_ and *k*_II_) and the cycle exiting inactivation of the catalysts (*k*_i_).

**Table 5:** Measured and Calculated TONs^a^

substrate	pH	catalyst	TON measured	TON calculated
ofloxacin	7.0	**2b**	1960	900
Reactive Black 5	7.0	**2b**	116	190
Orange II	7.0	**2a**	870	950
		**2b**	3370	3400
Safranine O	7.0	**2b**	800	2400
		**2c**	1630	4600
	11.0	**2b**	29	54
		**2c**	1650	3000

a Standard conditions: [Orange II] ∼5.0 × 10^–5^ M, [Safranine O] ∼3.0 × 10–6 M, [Reactive Black 5 or ofloxacin] ∼2.0 × 10^–5^ M, [H_2_O_2_] ∼ (1.0–5.0) × 10^–5^ M, [**2a**,**b**] ∼ (1.0–5.0) × 10^–8^ M, [**2c**] ∼ (0.1–1.0) × 10^–9^ M, reaction time ∼3 h, 0.01 M phosphate or carbonate buffer, 25 °C.

## Conclusions

This study provides a comprehensive characterization of the dynamics of the catalytic reactions (*k*_I_ and *k*_II_) and the inactivation reaction (*k*_i_) for NewTAMLs **2a**–**c**, thereby establishing multiple design-achieved benefits for the utility of oxidation systems. The results demonstrate that the C–H groups in the tail region of **2a** and **2b** function as KillSwitches that can be exploited to control catalyst lifetime independently of oxidative activity. The magnitude the KS effect increases strongly with pH between circumneutral conditions and pH 11–12 as progressive deprotonation of the tail center reveals the degradation susceptible ylide. Elimination of the KS was achieved through development of a new synthetic route converting **2b** to **2c** by quantitative methylation of the tail carbon center. Because **2c** contains no tail C–H bonds, the AC formed in **2c**/H_2_O_2_ processes cannot deprotonate to generate the carbanionic intermediates responsible for accelerated degradation in **2a** and **2b**. Consequently, the **2c**/H_2_O_2_ system proceeds to the next-fastest degradation pathway, which remains to be identified. That **2c** does decompose is important because achieving high health, environmental, and fairness performances in the pursuit of sustainable chemical products and processes mandates that Safe and Sustainable by Design chemicals possess finite lifetimes.^41^

Beyond defining the KS and its properties, this study also advances NT catalysis along the more familiar pathway in which optimization of technical and cost performances drives the attractiveness of applications. Suppression of the KS leaves the key catalytic reactions largely unchanged. Thus, although **2c**/H_2_O_2_ exhibits oxidative aggression comparable to that of both **2a**/H_2_O_2_ and **2b**/H_2_O_2_, its substantially extended lifetime affords distinctly superior overall technical performance across the full working pH range, with the advantages increasing strongly with pH. NT **2c** is substantially more attractive than **2a** and **2b** for oxidation processes conducted under basic conditions.

## Supplementary Material



## Notes

Figures, including ORTEP diagram of [PPh_4_]­[**2c**]_EtOH_, rate vs [Fe^III^]_t_; all 3D plots within pH 6.0–12.5 and 3D mesh plots for catalyst **2c** at 40–60 °C at pH 7.0; the incomplete bleaching of Orange II for catalyst **2c** at 40–60 °C at pH 7.0; Eyring plots for catalyst **2c**; dependence of *k*_i_ values on H_2_O_2_ at pH 8.0; and dependence *k*_i_–*k*_i_^0^ on pH for catalyst **2b**. Tables including experimental details regarding the synthesis of **2c**, rate constants (*k*_I_, *k*_II_, and *k*_i_) for the **2**-catalyzed degradation of Orange II and Safranine O by H_2_O_2_ at pH 6–12 under different temperatures, and the conditions used (PDF)
