# Supplementary material for: A Programmable KillSwitch in NewTAML Catalysts Decouples Oxidative Reactivity from Catalyst Lifetime
Source: J Am Chem Soc. 2026 Oct 7;148(41):44360–71. doi: 10.1021/jacs.6c11419 (PMC13693390; doi:10.1021/jacs.6c11419)
Supplement: Supplementary file 1 [file ja6c11419_si_001.pdf]

Supporting Information

A Programmable KillSwitch in NewTAML Catalysts Decouples Oxidative Reactivity from Catalyst Lifetime

Debojyoti Chakraborty, [a] Minerva G. Schafer, [a] Xiaowei Ma,[a] Chris Birch,[b] Carlos Felipe Mejía,[c] Rubén A. Toscano,[c] Ronan Le Lagadec,[c] Maxime A. Siegler,[d] Kevin Noonan,*[b] Alexander D. Ryabov,* [a] Terrence J. Collins,* [a]

[a] Institute of Green Science (IGS), Carnegie Mellon University, Pittsburgh, Pennsylvania, 15213, USA.

[b] Carnegie Mellon University, Pittsburgh, Pennsylvania, 15213, USA.

[c]Instituto de Química, Universidad Nacional Autónoma de México, Circuito Exterior s/n, Ciudad Universitaria, Ciudad de México 04510, Mexico.

[d]Department of Chemistry, The Johns Hopkins University, Baltimore, Maryland 21218, United States.

Determination of k_i for 2c at 25 ºC. The rate constants k_I and k_{II} at 25 – 60 ºC were obtained by fitting the kinetic data shown in 3D Figure S4 to eq 10. The rate constants k_i were calculated using eq 13 applying the regime of incomplete bleaching of Orange II at 40 – 60 ºC. Typical kinetic traces for determining S_∞ are shown in Figure S5. The Eyring plots for k_I, k_{II} and k_i are shown in Figure S6.

Figures:

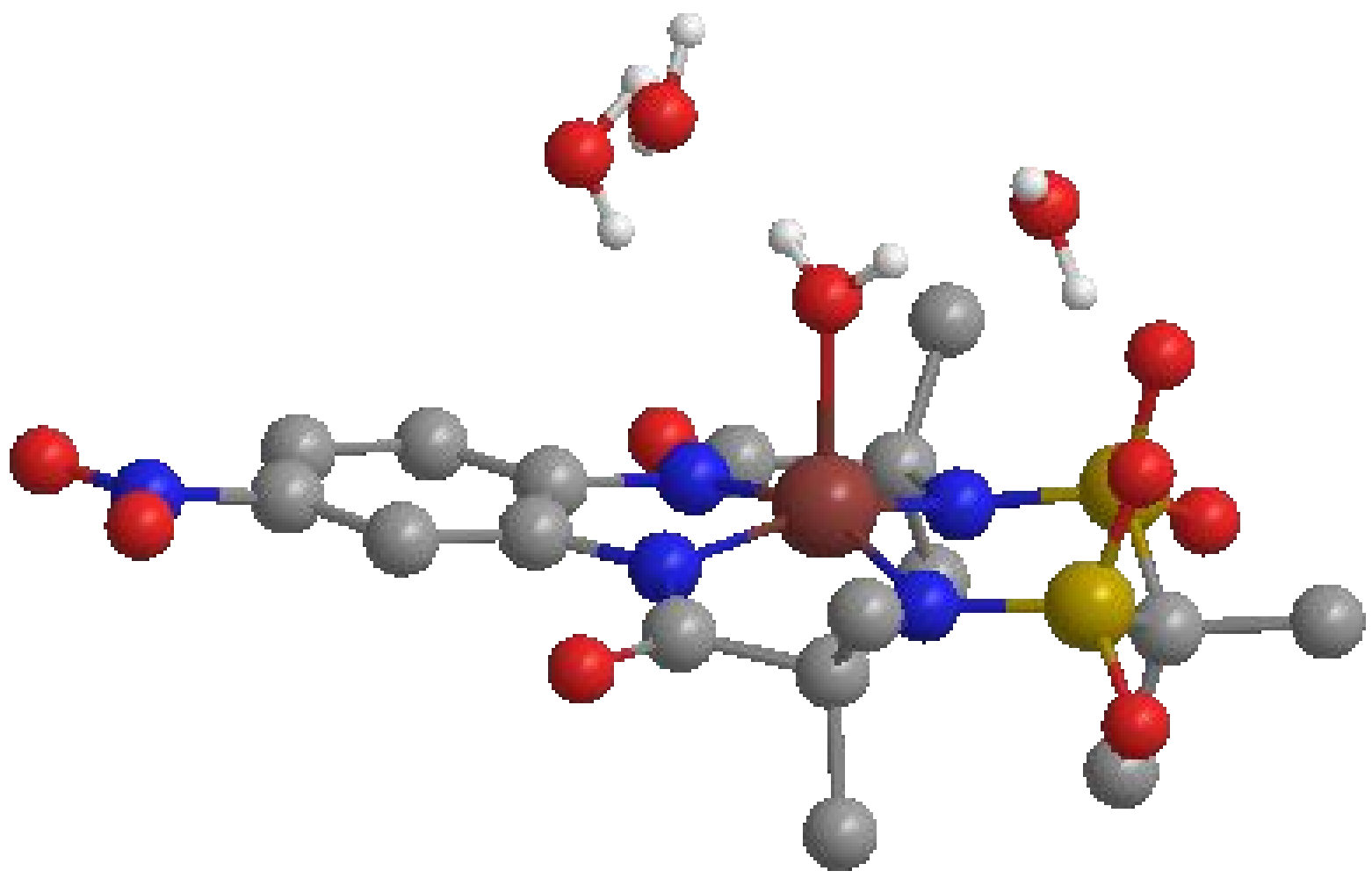

Figure S1. ORTEP diagram of [PPh_4][**2c**]$_{EtOH}$ showing all three water molecules in the unit cell of the [**2c**][PPh_4] crystals grown from H_2O/EtOH.

As presented in Table 1, the key bond lengths and bond angles of [PPh_4][**2c**]$_{EtOH}$ and [PPh_4][**2c**]$_{MeCN}$ vary insignificantly. The differences concern the number of water molecules co-crystallized with **2c**. Three water molecules surround [PPh_4][**2c**]$_{EtOH}$, whereas one is seen in [PPh_4][**2c**]$_{MeCN}$. One water molecule, which is hydrogen bonded with other atoms, is shown for [PPh_4][**2c**]$_{EtOH}$ in Figure 1. There are hydrogen bonds with the axial aqua ligand and the oxygen of the O=S1 bond. The corresponding H···O separations equal 1.899 and 1.808 Å, respectively. Both are noticeably shorter than the sum of the van der Waals radii of H and O atoms (2.77 Å). Two other distal H_2O

molecules of the unit cell of [PPh_4][**2c**]$_{EtOH}$ (Figure S1) have no close contact with the **2c** anion.

The major difference between [PPh_4][**2c**]$_{EtOH}$ and [PPh_4][**2c**]$_{MeCN}$ is the location of the hydrogen bonded water molecule. Acetonitrile, as a more hydrophobic co-solvent than ethanol, moves co-crystallized water to a more hydrophobic part of the anion, closer to the aromatic ring, keeping the O···H separation of 1.893 Å. Compared to EtOH, acetonitrile as a co-solvent for crystal growth (*i*) minimizes a number of water molecules in the unit cell and (*ii*) moves the hydrogen bonded water to a more hydrophobic part of the TAML anion. The overall geometry of **2c** within [PPh_4][**2c**]$_{EtOH}$ and [PPh_4][**2c**]$_{MeCN}$ does not seem to be affected.

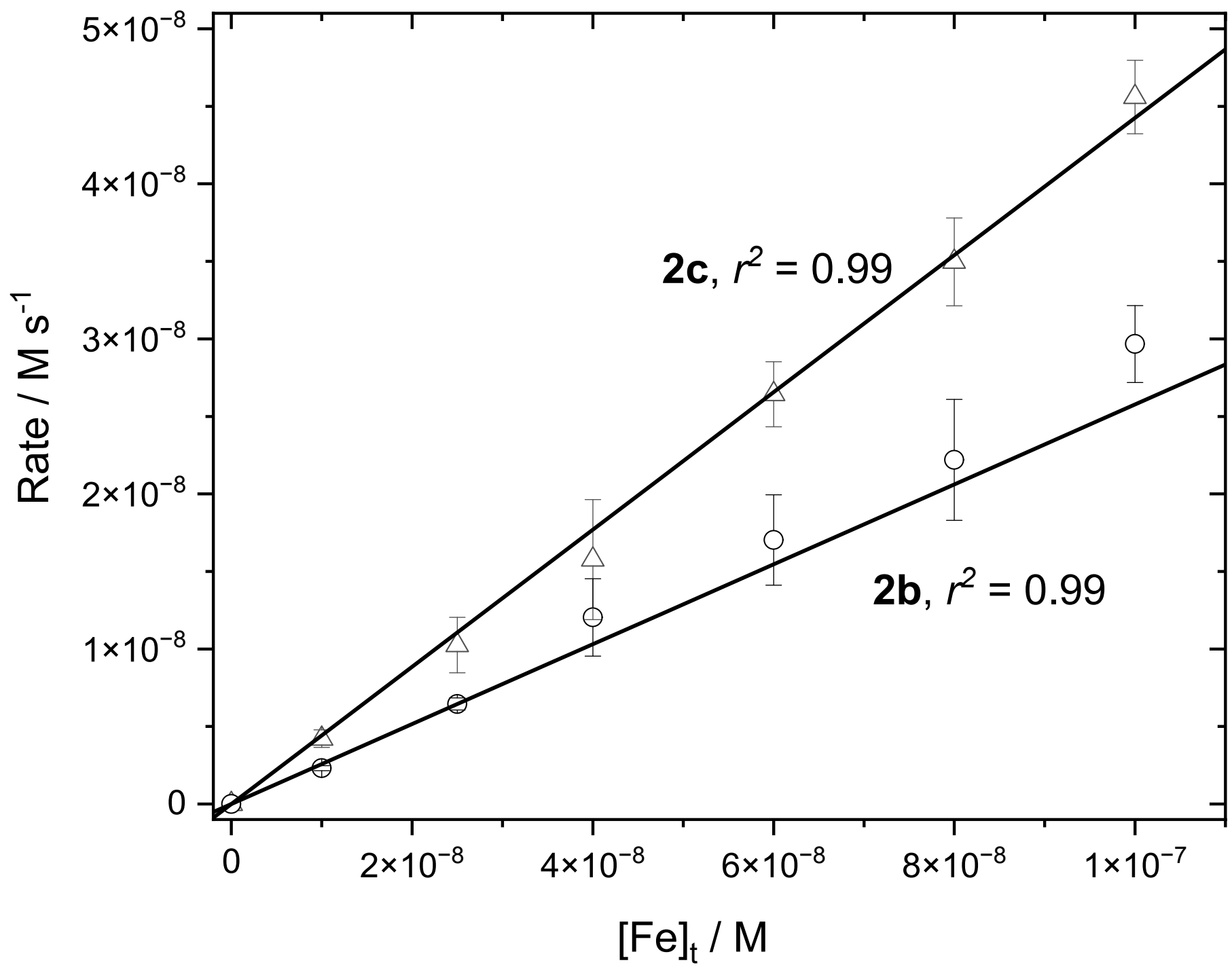

Figure S2. Initial rates of catalyzed by **2b** and **2c** oxidation of Orange II (2.5×10^{-5} M) by H_2O_2 (1.0×10^{-3} M) as a function of [**2b**] and [**2c**] at pH 7 (0.01 M phosphate) and 25 ^{o}C.

pH	**2a**	**2b**	**2c**
Orange II			
6.0	3D plot of initial rates at pH 6.0, [**2a**] = 100 nM 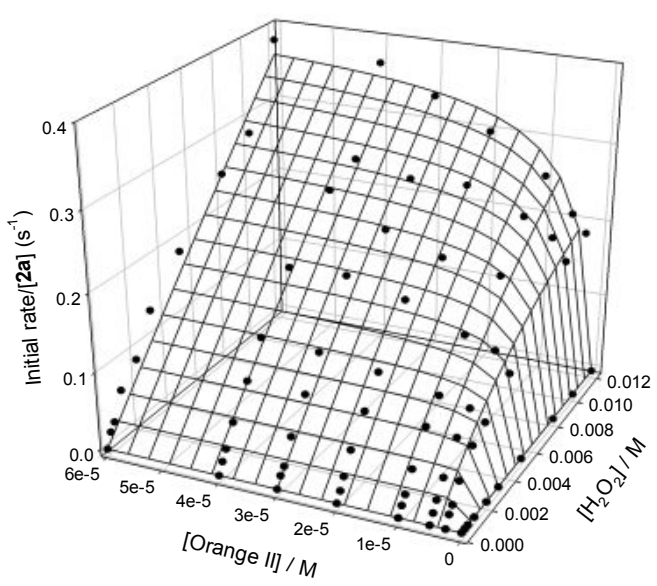	3D plot of initial rates at pH 6.0, [**2b**] = 75 nM 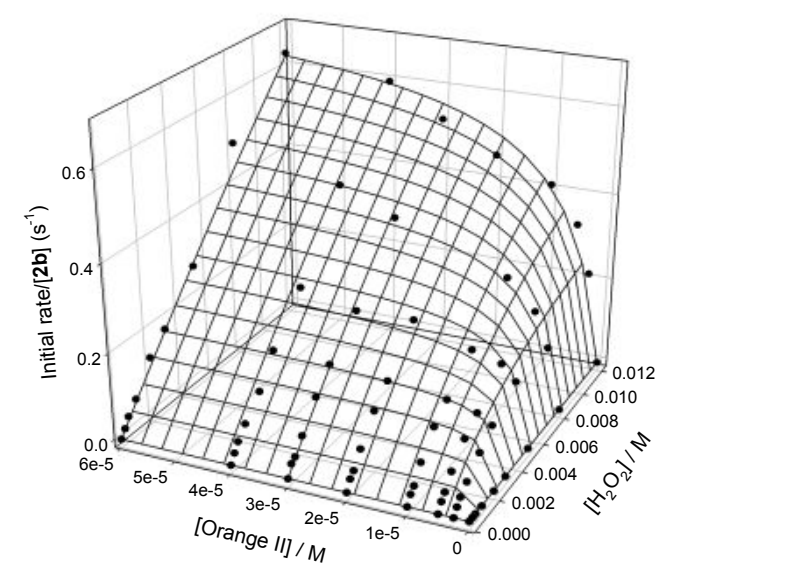	3D plot of initial rates at pH 6.0, [**2c**] = 100 nM 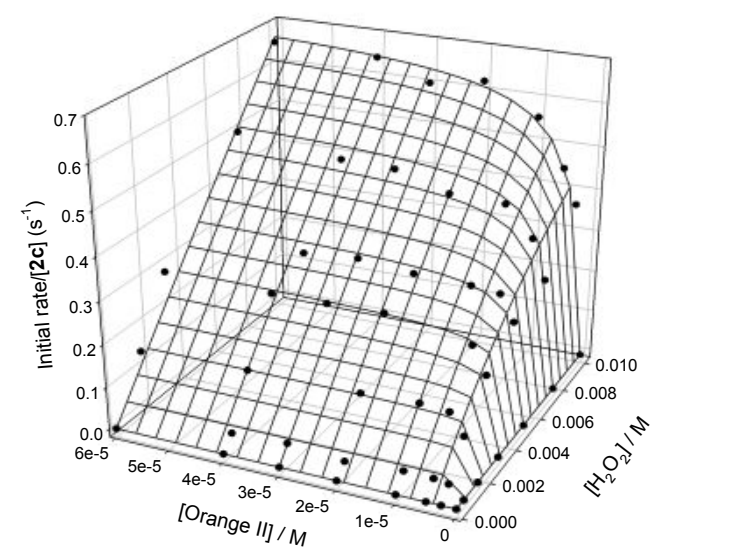
6.5	3D plot of initial rates at pH 6.5, [**2a**] = 80 nM 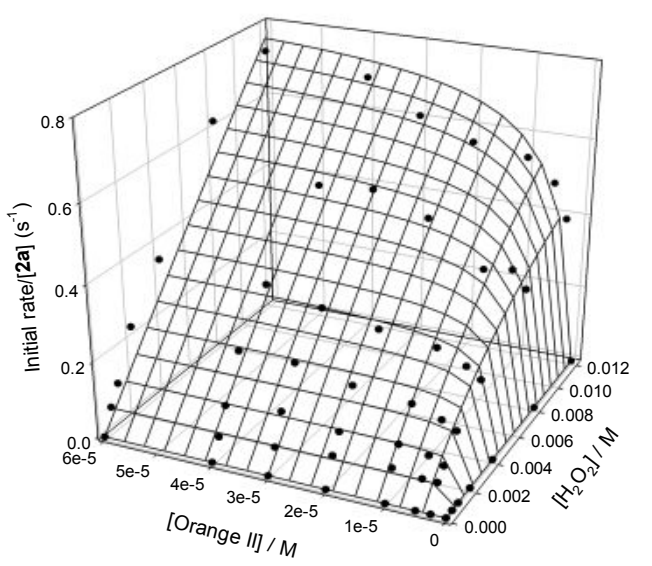	3D plot of initial rates at pH 6.5, [**2b**] = 40 nM 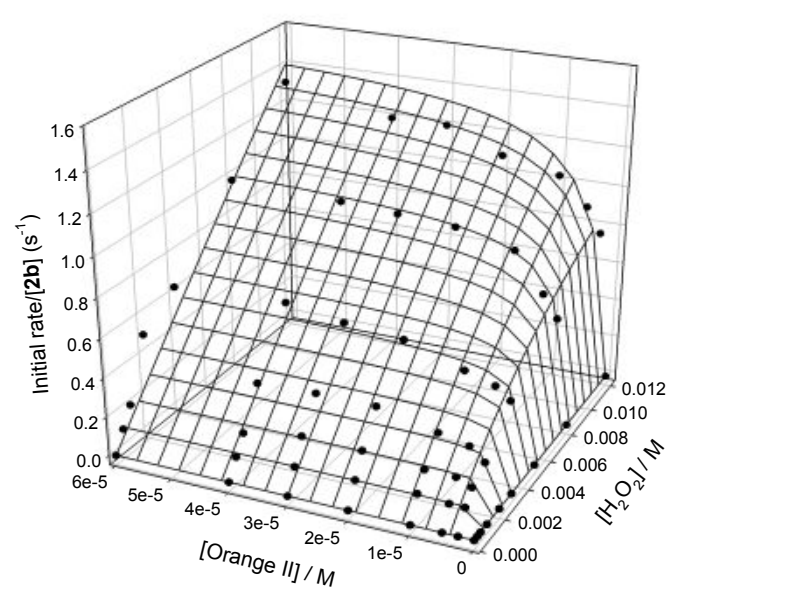	3D plot of initial rates at pH 6.5, [**2c**] = 50 nM 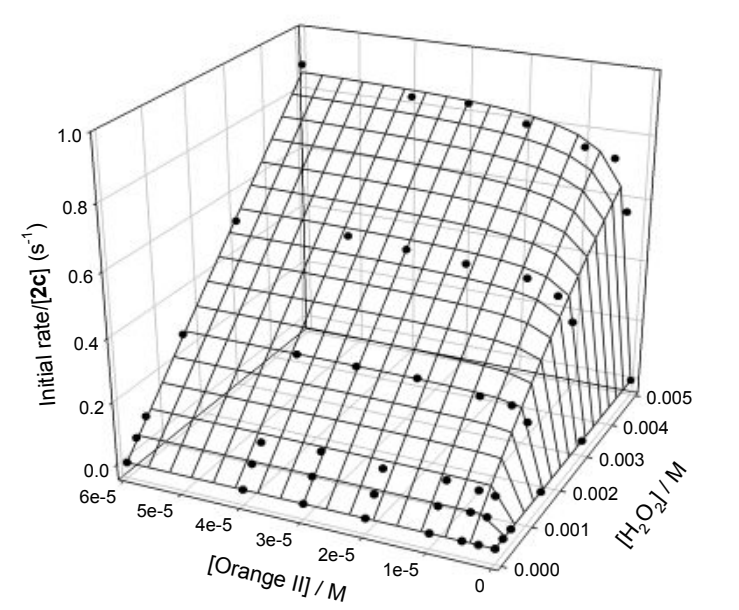
7.0	3D plot of initial rates at pH 7.0, [**2a**] = 50 nM 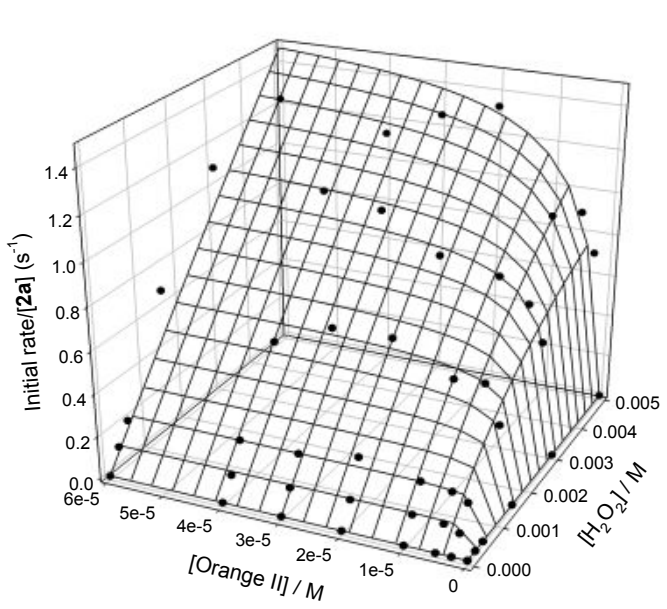	3D plot of initial rates at pH 7.0, [**2b**] = 50 nM 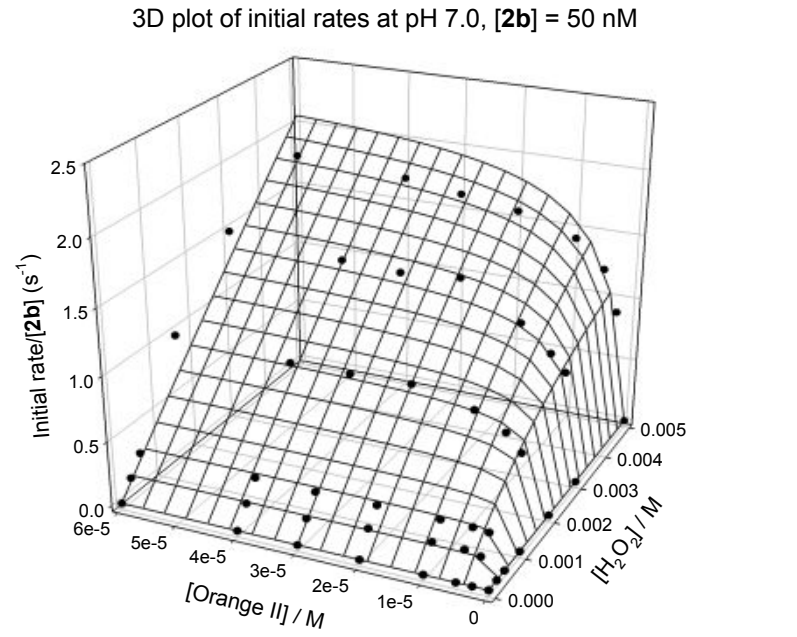	3D plot of initial rates at pH 7.0, [**2c**] = 50 nM 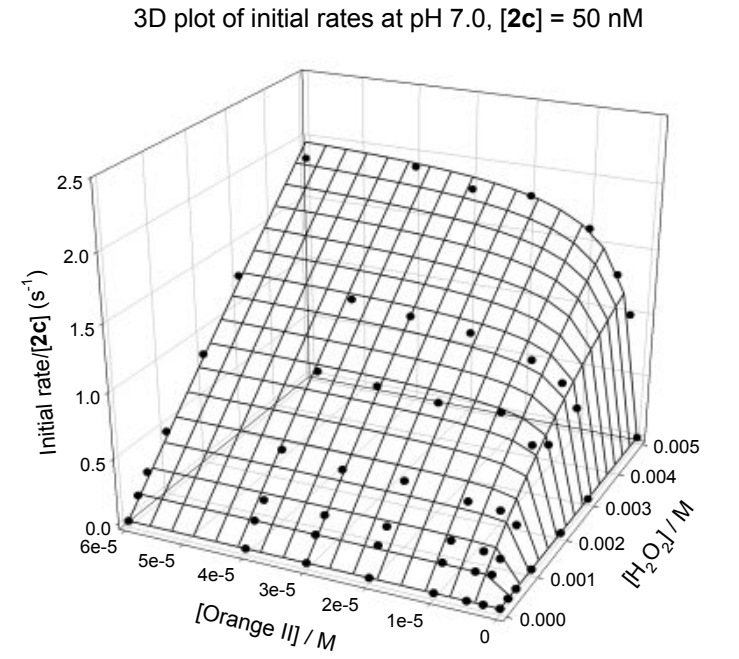
7.5	3D plot of initial rates at pH 7.5, [**2a**] = 50 nM 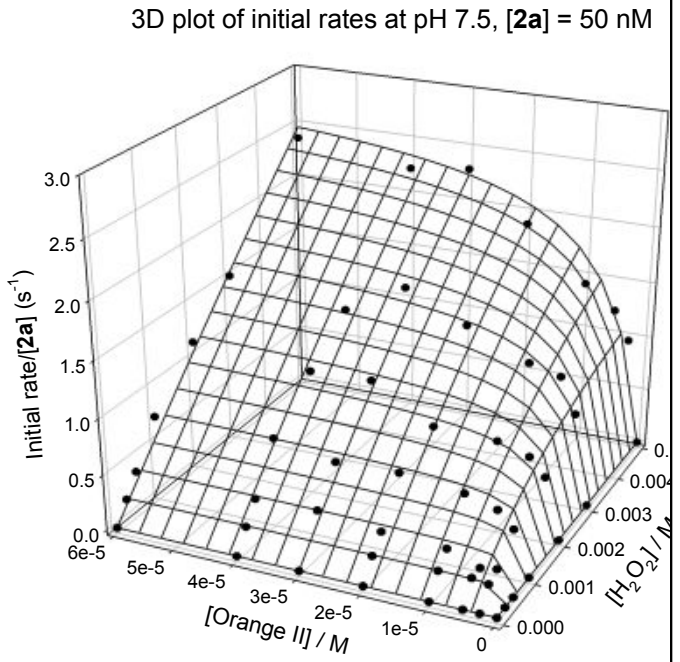	3D plot of initial rates at pH 7.5, [**2b**] = 20 nM 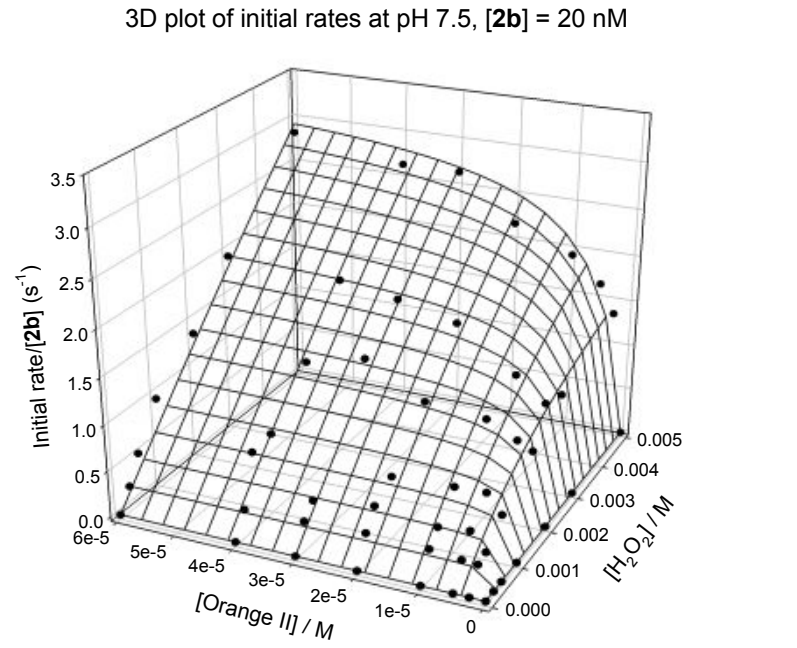	3D plot of initial rates at pH 7.5, [**2c**] = 20 nM 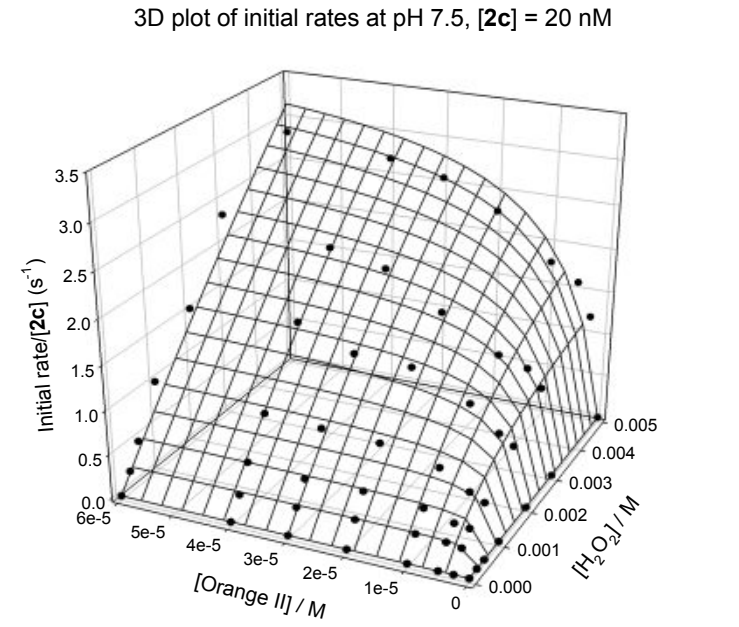

8.0	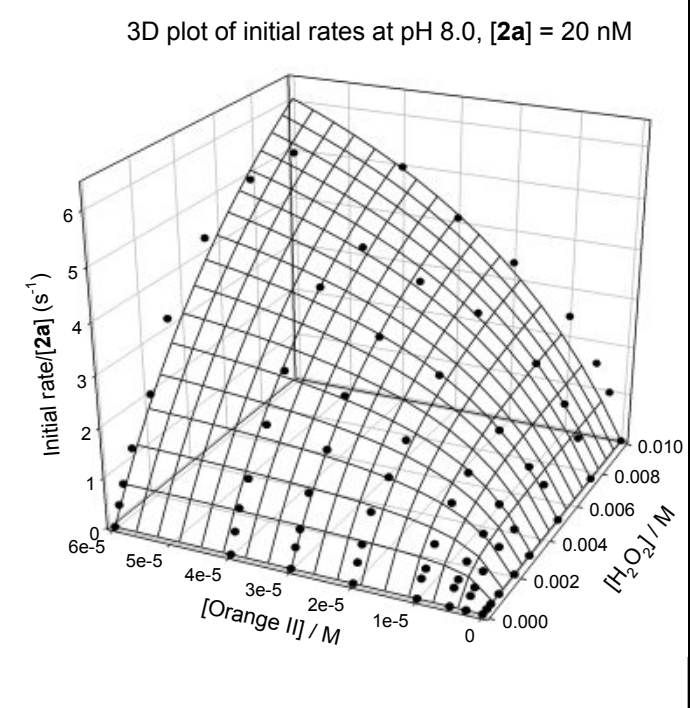	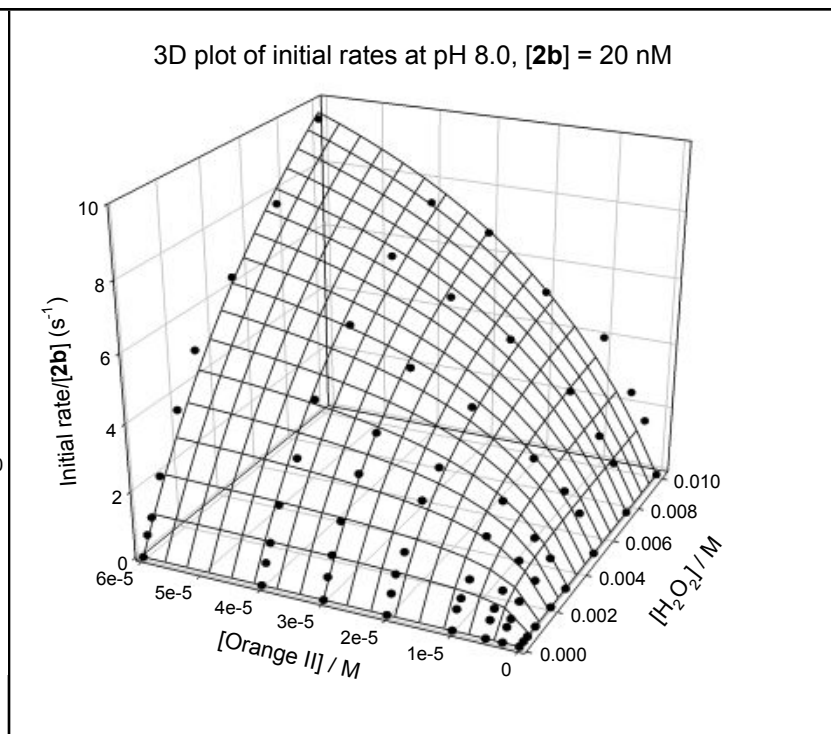	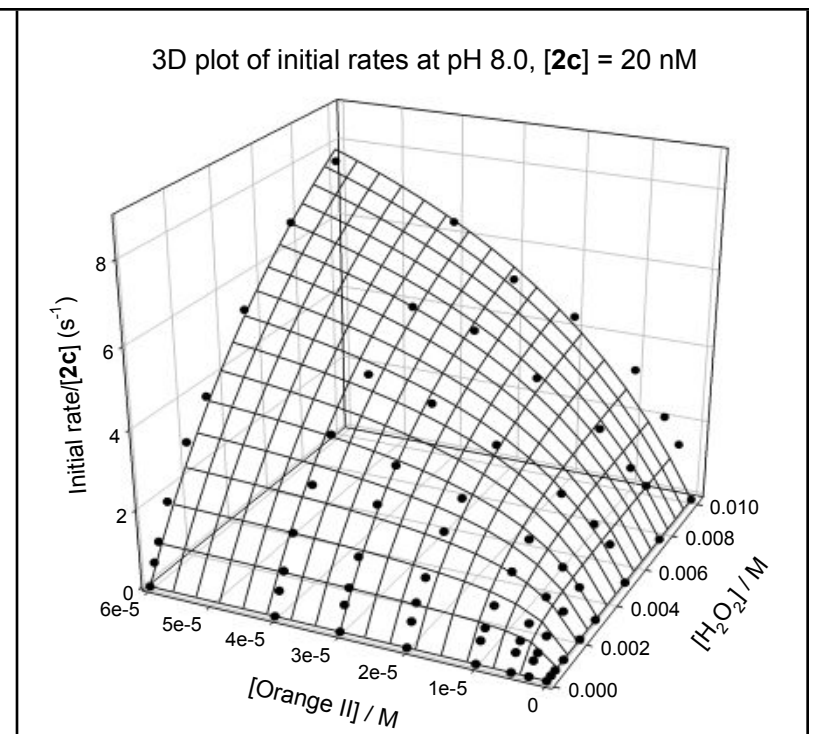
9.0	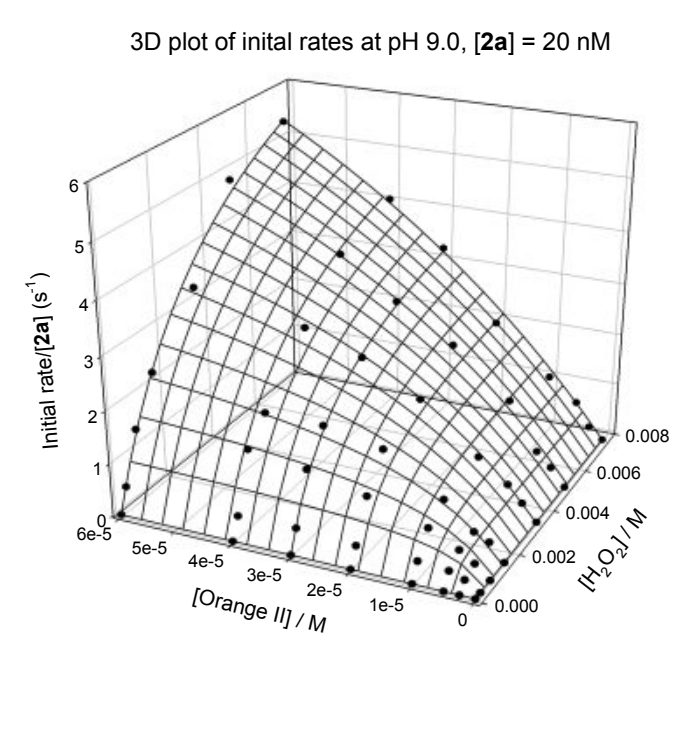	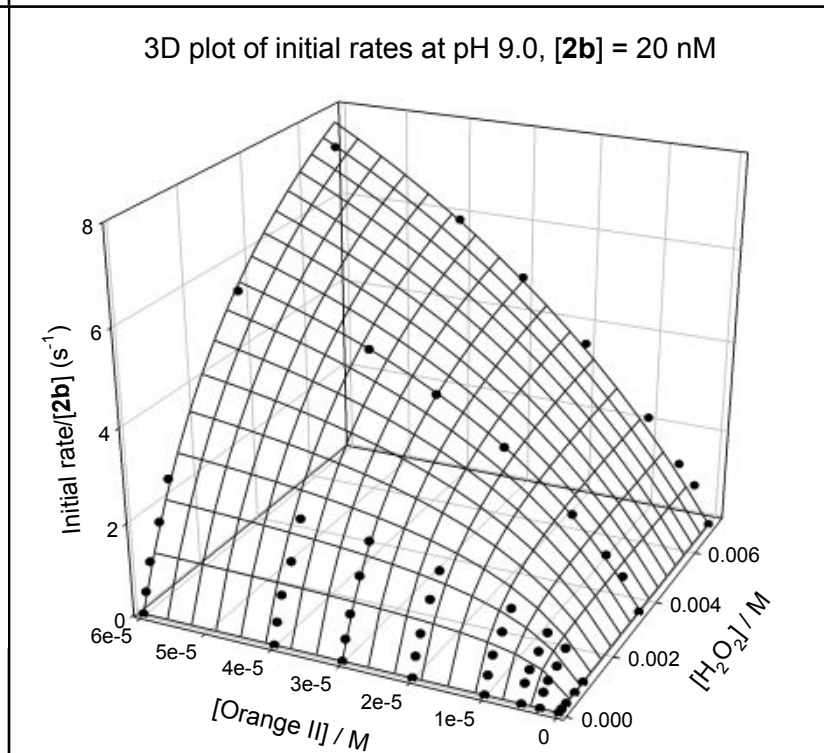	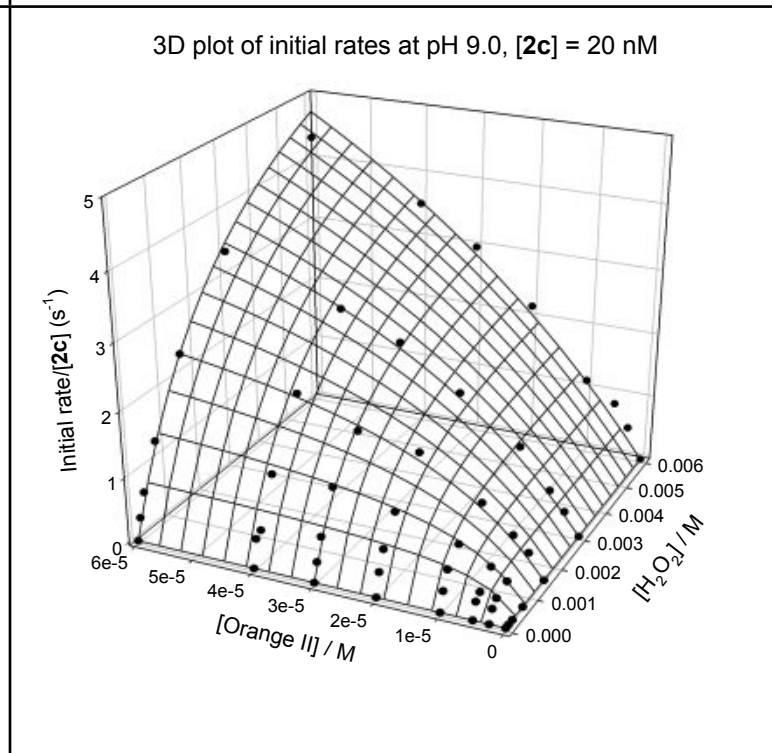
10.0	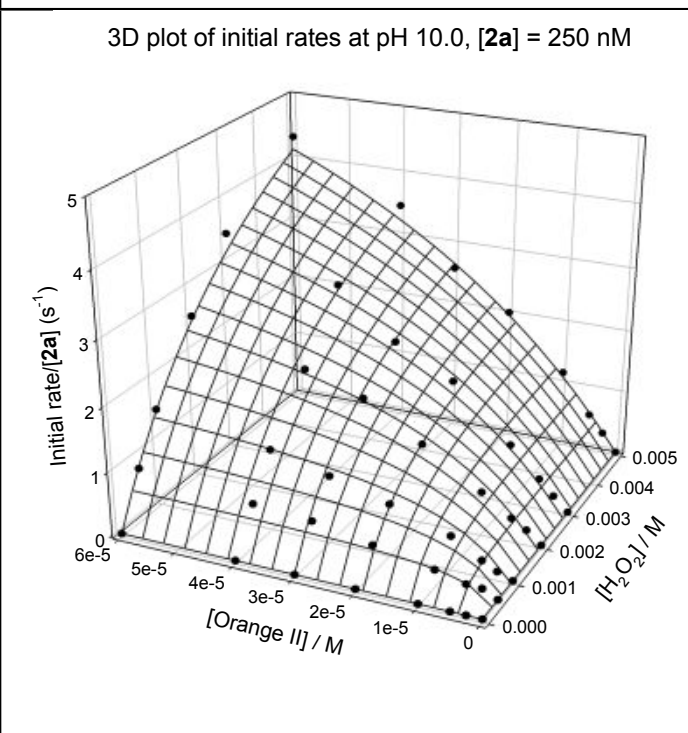	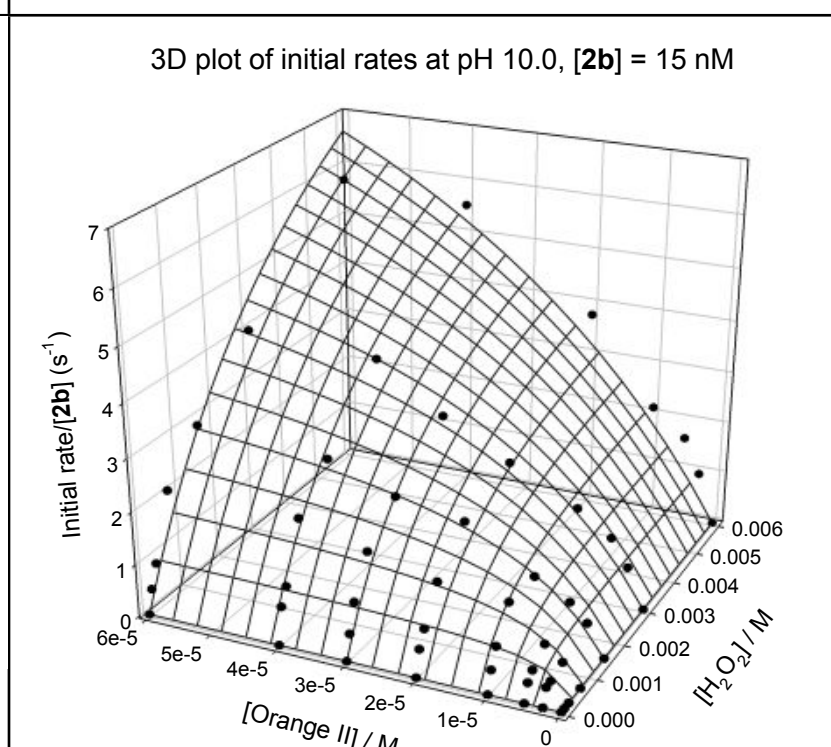	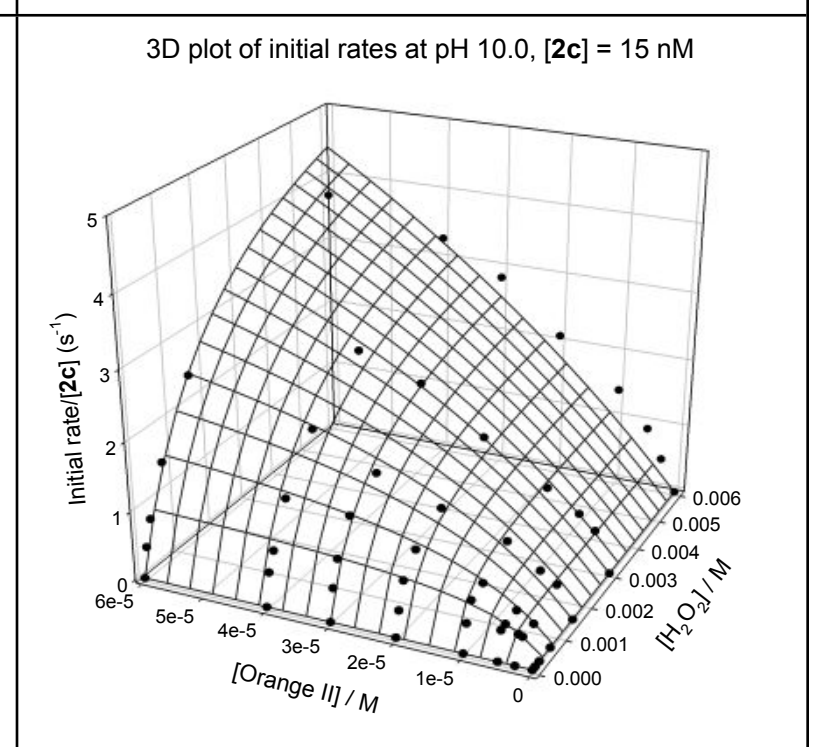
11.0	na	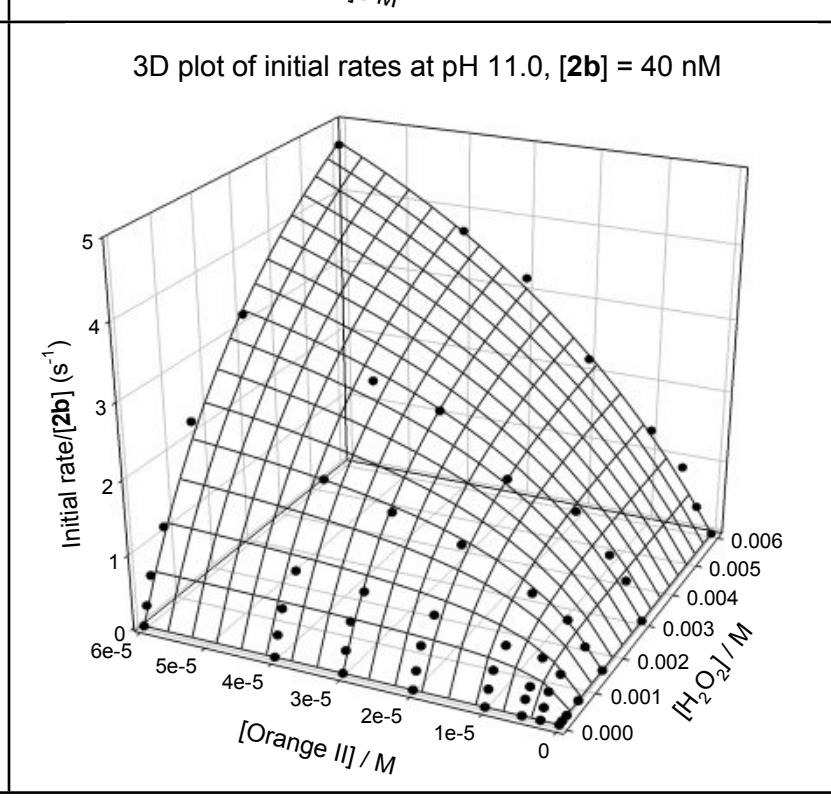	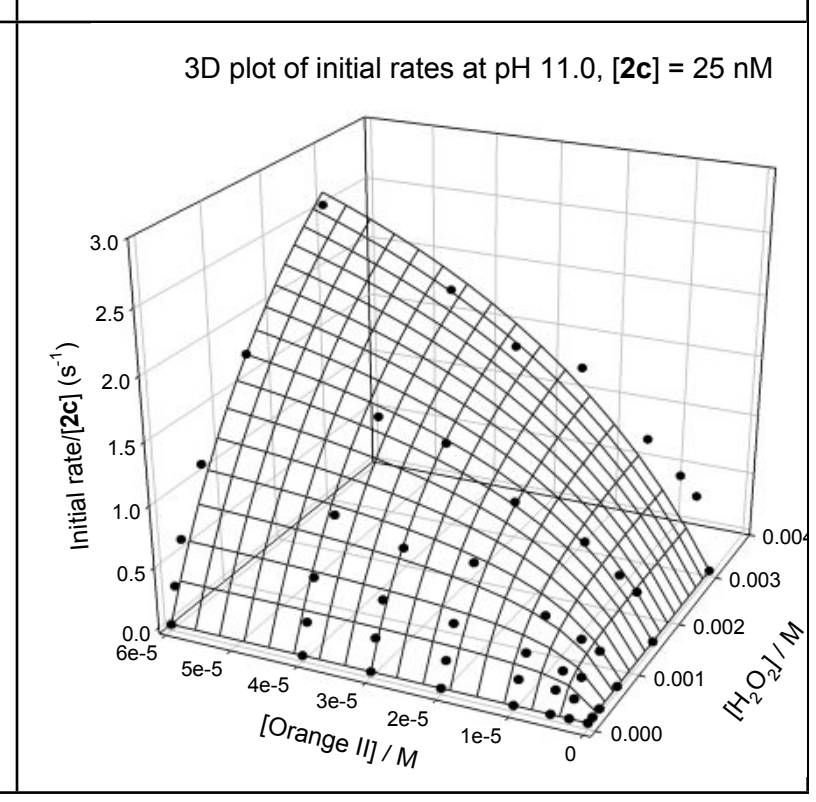

11.5	na	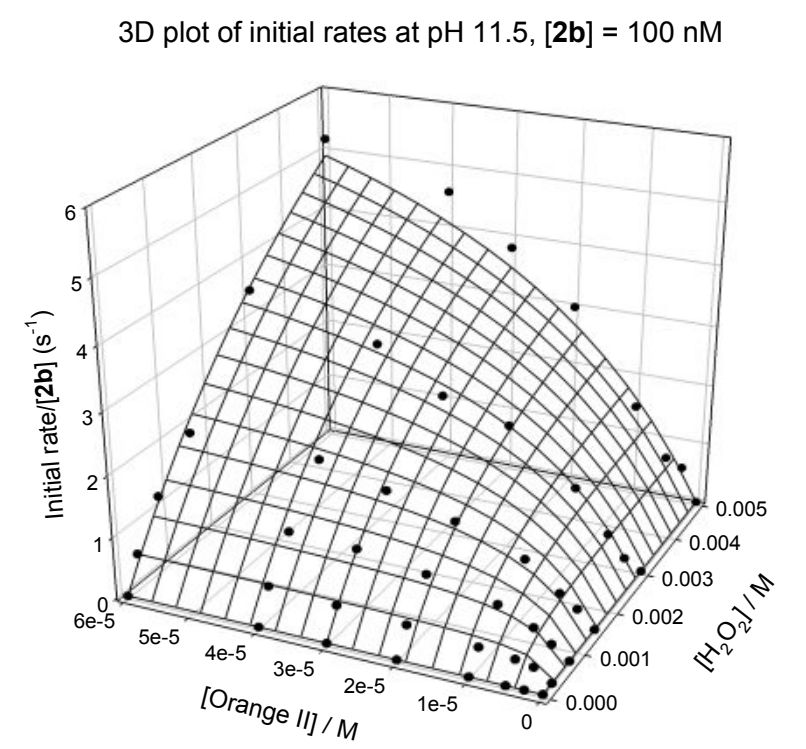	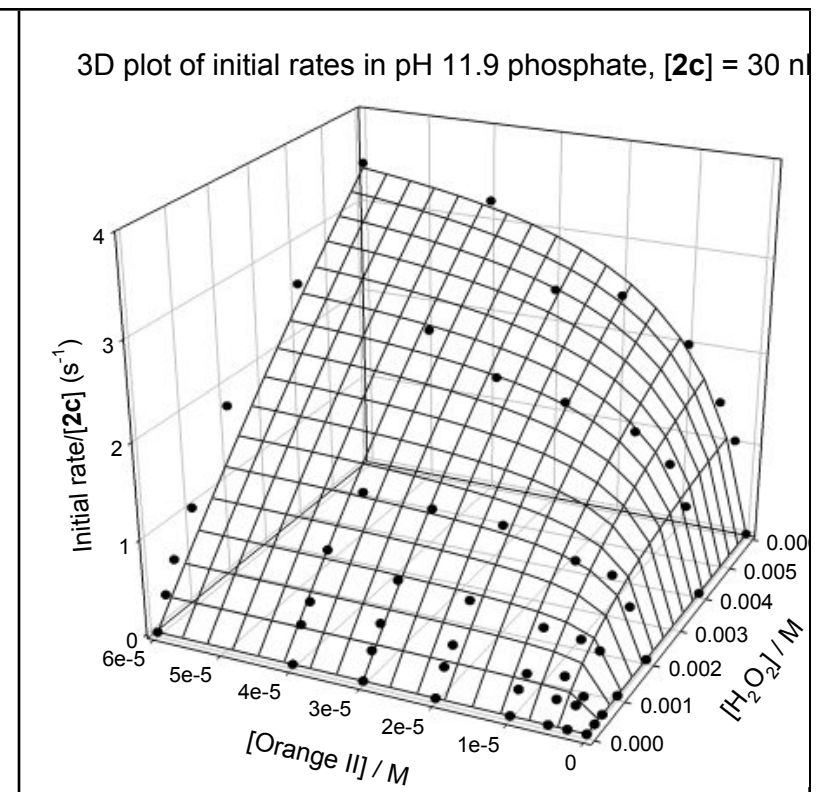
11.9	na	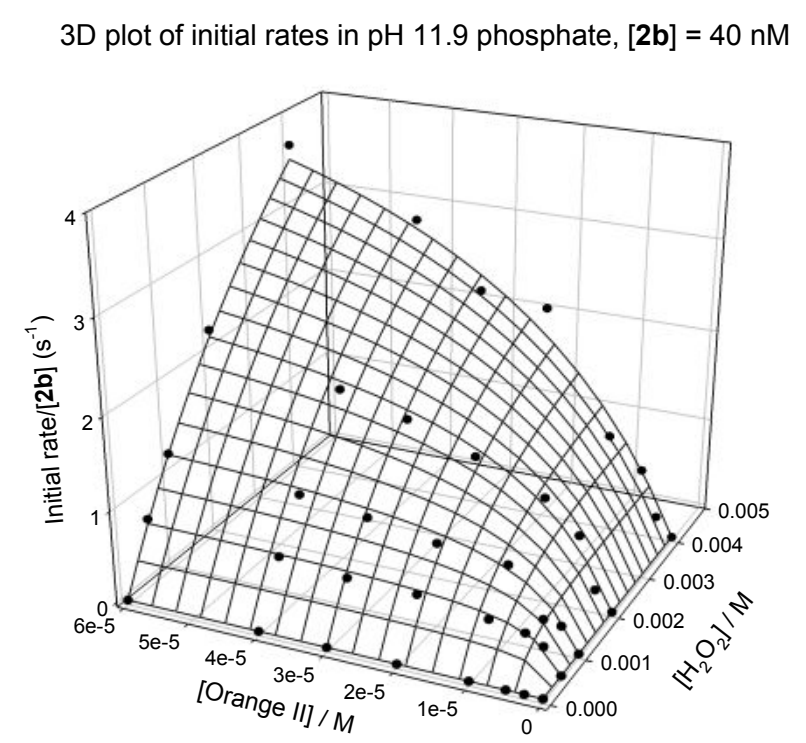	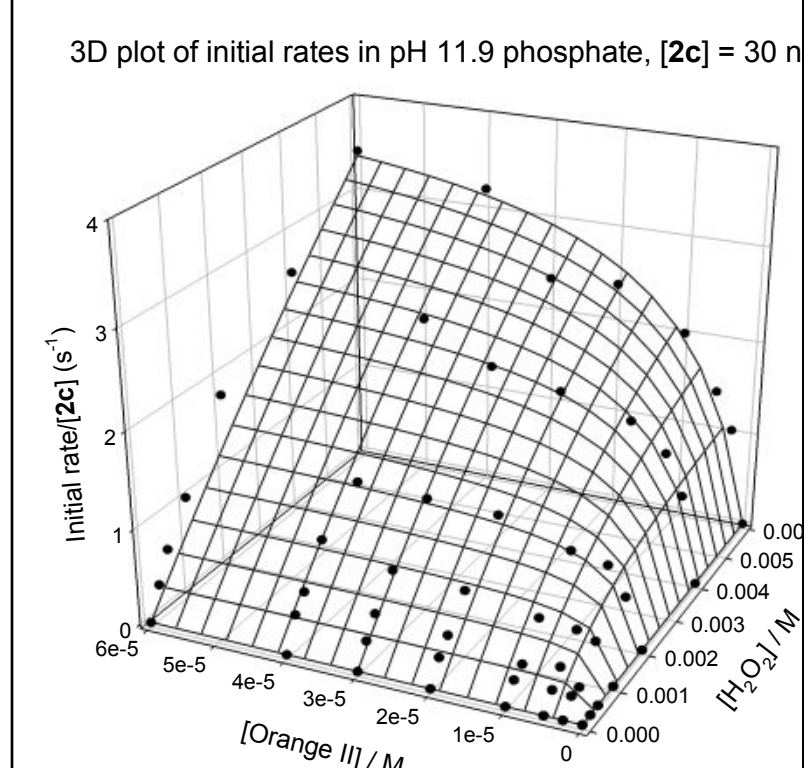
12.5	na	na	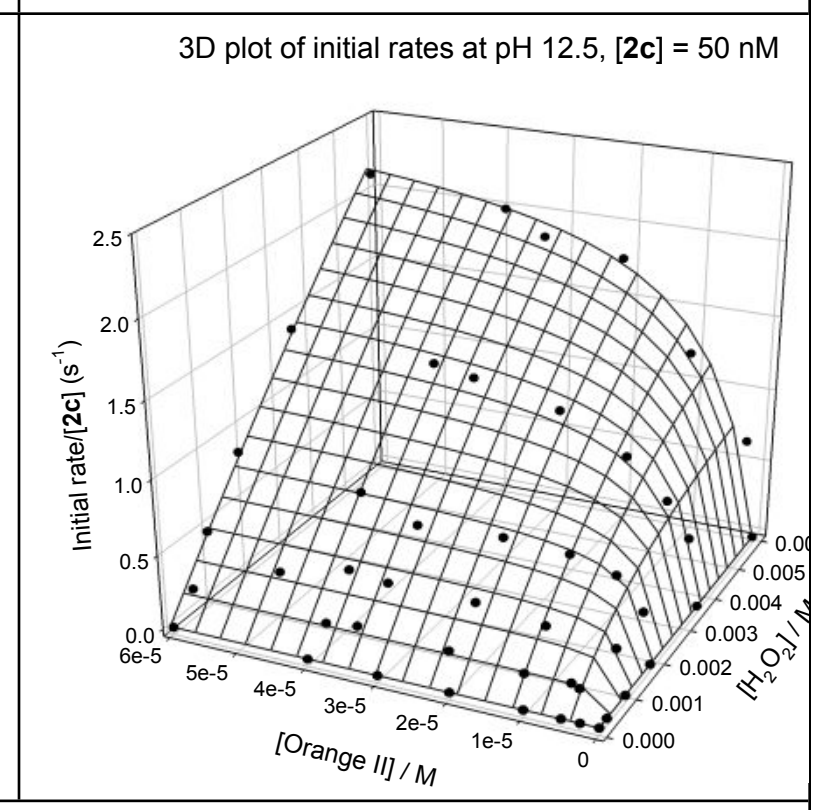
Safranine O			
7.0	na	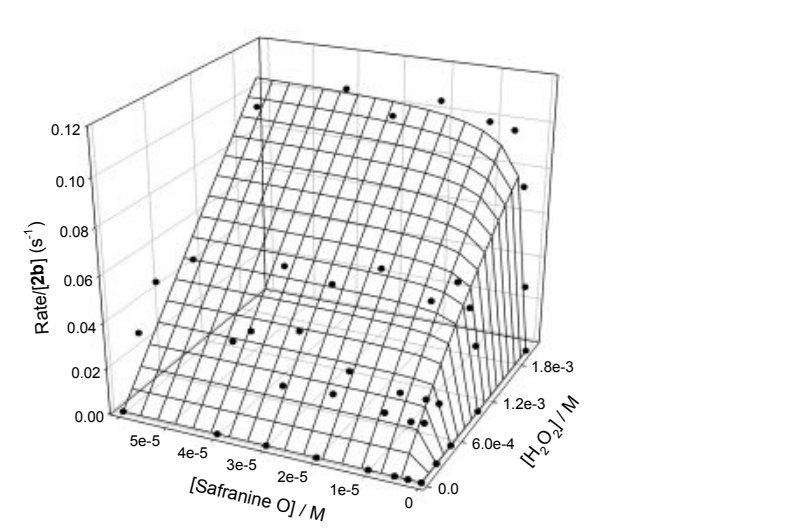	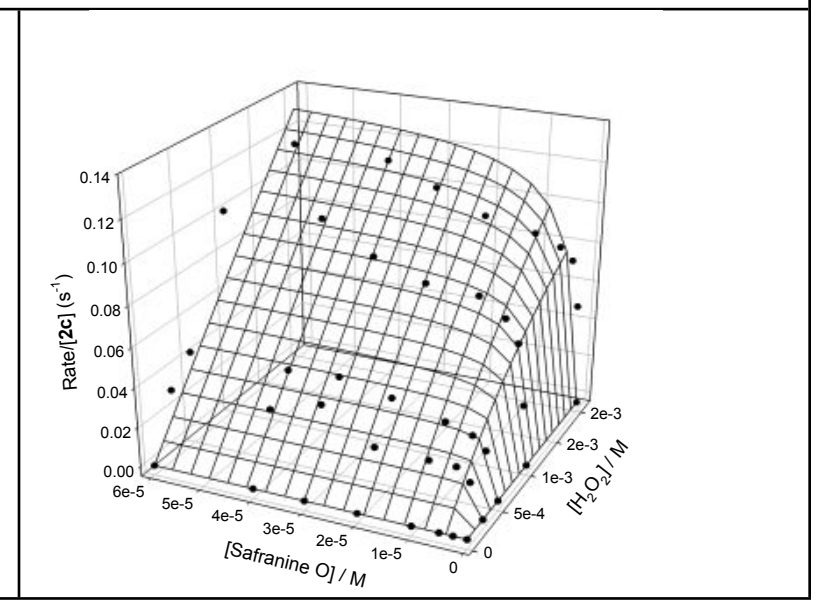

Figure S3. 3D plots of initial rates ($-d$[S]/dt) of oxidation of Orange **II** and Safranine O dyes by H_2O_2 catalyzed by **2a-c** at pH 6.0-12.5 in 0.01 M phosphate (pH 6.0-8.0, >

11.5) or carbonate (pH 9.0-11.5) buffers and 25 °C as functions of concentrations of H_2O_2 and Orange II.

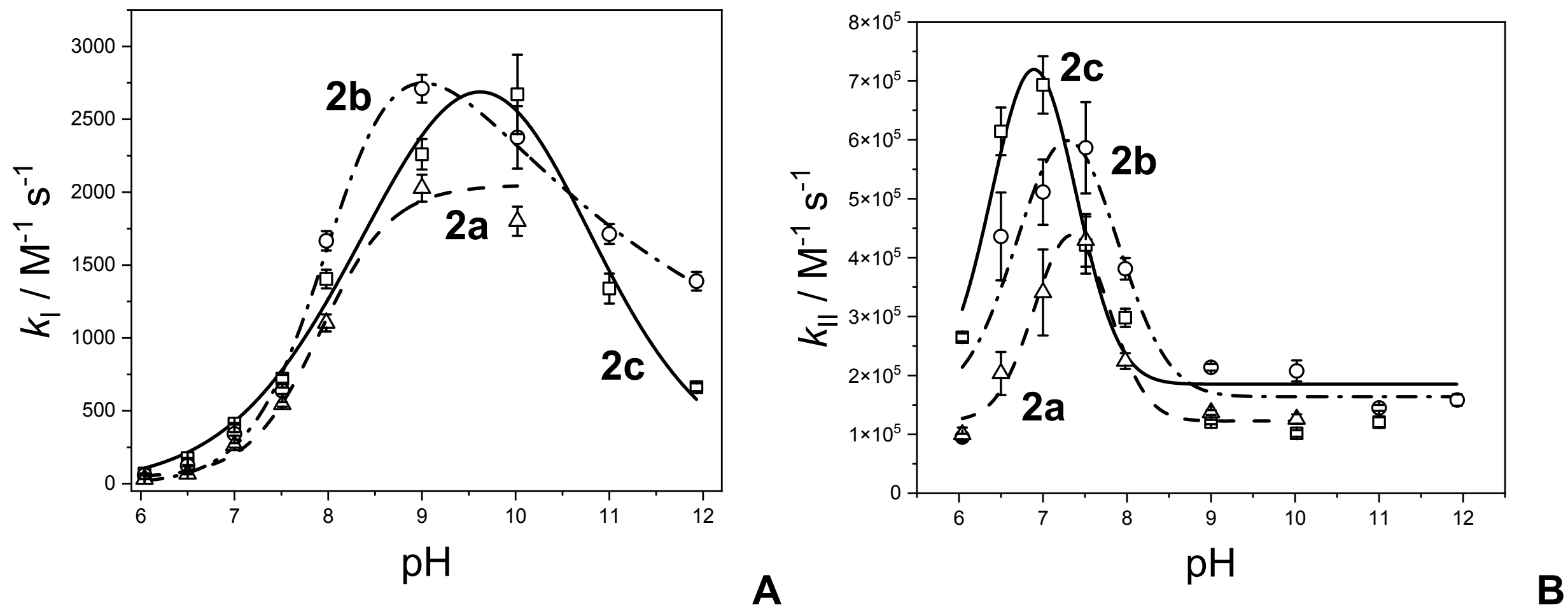

Figure S4. pH profiles for the rate constants k_I (**A**) and k_{II} (**B**) at 25 ºC. The instability of **2a** at pH > 10 does not permit calculation of k_I and k_{II} because of its low catalytic activity (see below).

The bell-shaped k_I pH profiles in Figure S4A result from deprotonation of reactive species at pH 6 – 12 (eq 4 for **2** plus $H_2O_2 \rightleftarrows HO_2^- + H^+$). Importantly, the absolute value of k_{II} for **2c** at pH 7.0 of 7×10^5 M^{-1} s^{-1} highlights its remarkable and frontier reactivity compared with other iron TAML activators.

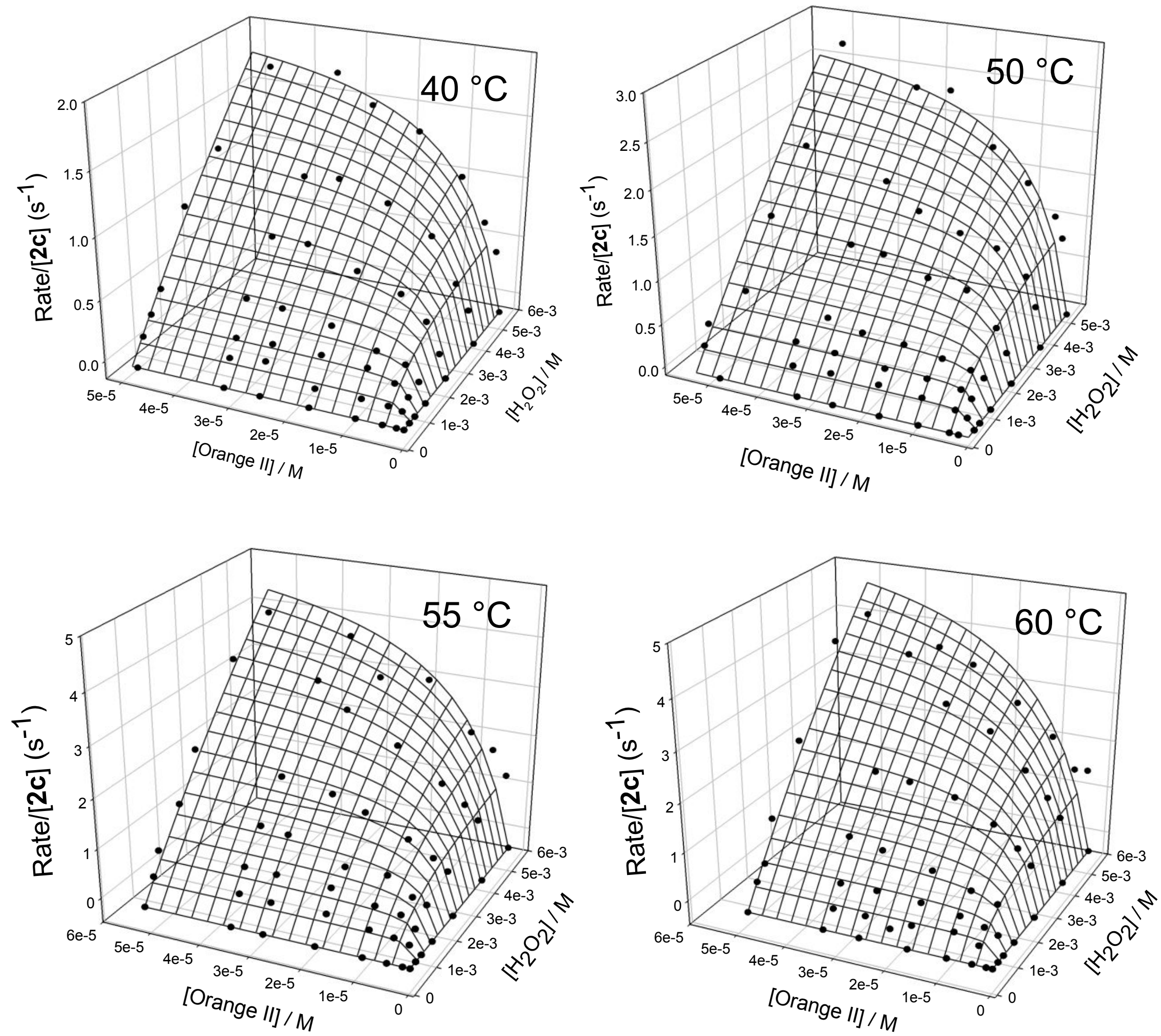

Figure S5. 3D plots showing dependencies of initial rates of Orange II bleaching by H_2O_2 catalyzed by **2c** (2.0×10^{-8} M at 40 and 50 °C); 1.5×10^{-8} M at 55 and 60 °C. [H_2O_2] 0 - 7.5×10^{-3} M; [Orange II] 0 - 5.5×10^{-5} M; pH 7.0, 0.01 M phosphate, 40 – 60 ºC.

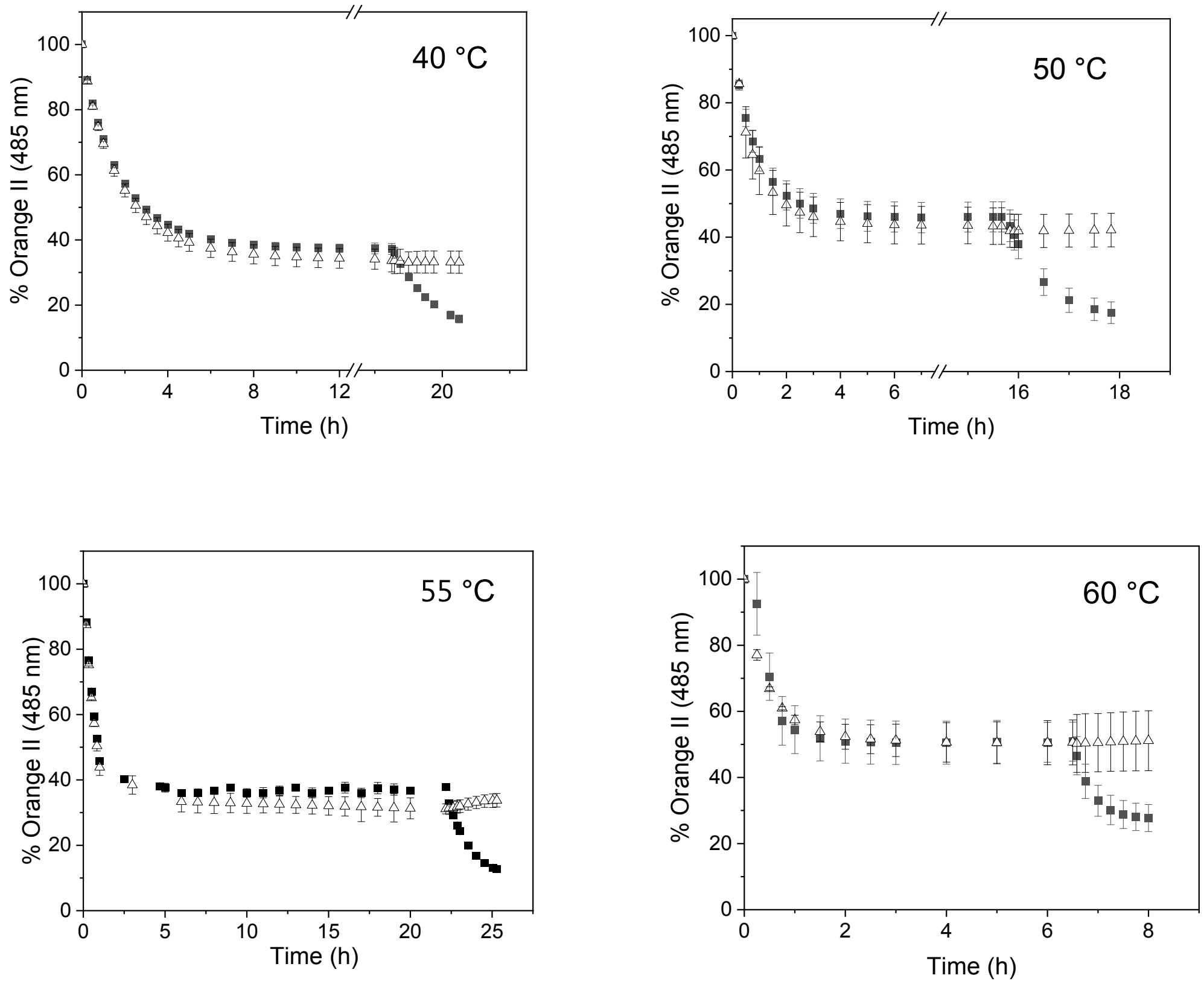

Figure S6. Kinetic traces of incomplete bleaching of Orange II (5.0×10^{-5} M) by H_2O_2 (1.0×10^{-3} M) catalyzed by **2c** (1.0×10^{-8} M) at 40 – 60 ºC and pH 7 (0.01 M phosphate) used for determining S_∞ and k_i via eq 13. White triangles (△): addition of second aliquot of H_2O_2. Solid squares (■): addition of second aliquot of **2c**. Second aliquots for H_2O_2 or **2c** were added once the bleaching rate plateaued significantly.

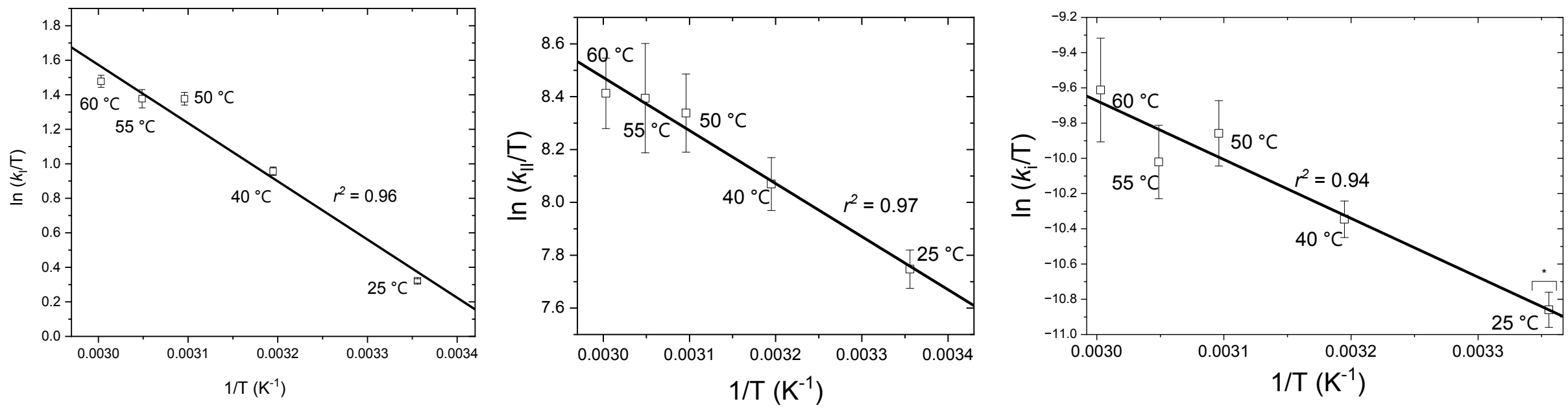

Figure S7. Eyring plots for the rate constants k_{I}, k_{II} and k_{i} for the **2c**-catalyzed oxidation of Orange II at pH 7 (0.01 M phosphate). The plot for k_{i} was used for estimating this rate constant at 25 ºC.

Equation used: $\ln\left(\frac{k}{T}\right) = \left(-\frac{\Delta H^{\neq}}{R}\right)\left(\frac{1}{\mathrm{T}}\right) + \left[\ln\left(\frac{k_{\mathrm{B}}}{h}\right) + \left(\frac{\Delta S^{\neq}}{R}\right)\right]$

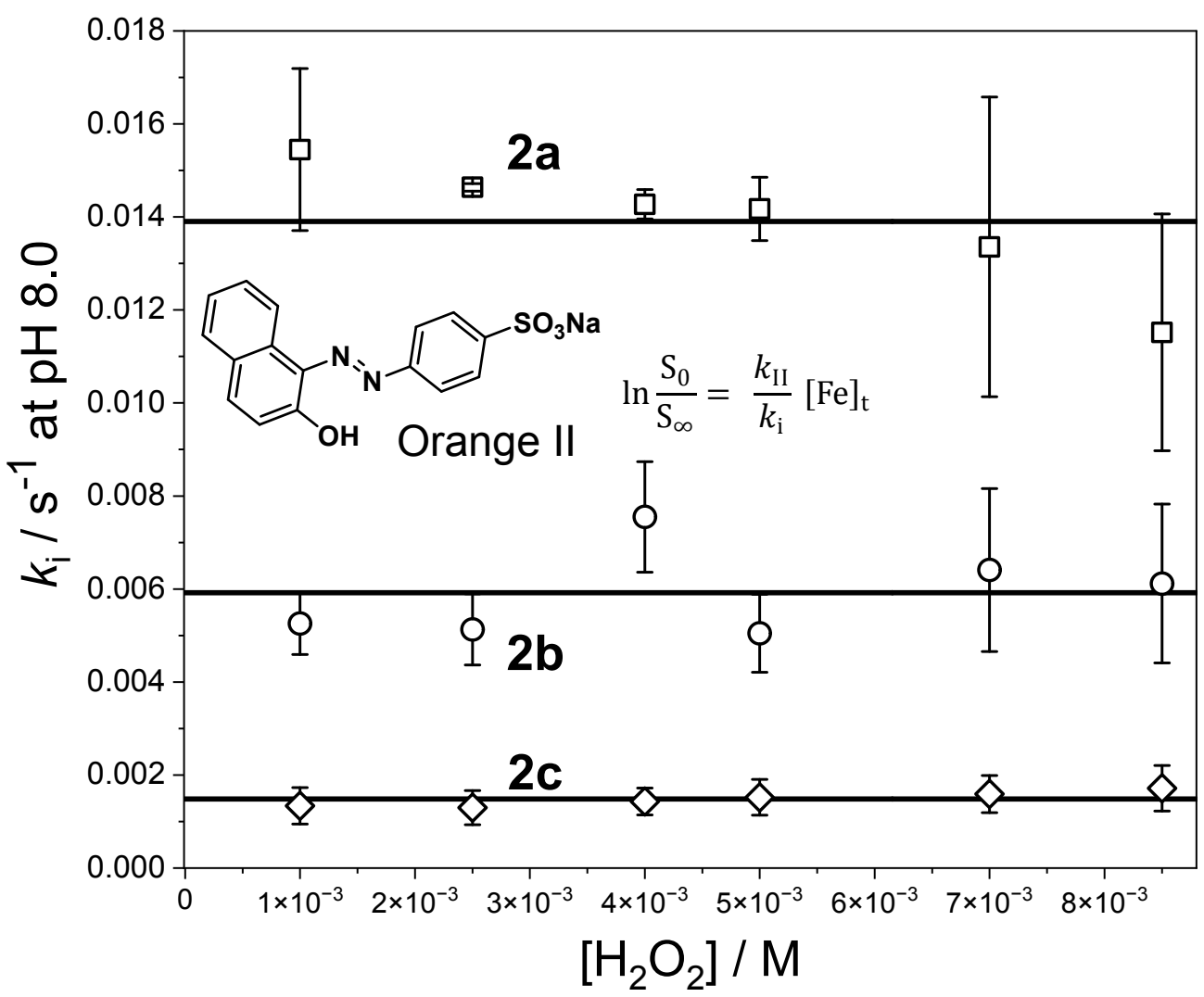

Figure S8. Rate constants k_i against [H_2O_2] for **2a** (1.0×10^{-8} M), **2b** (2.5×10^{-9} M), **2c** (1.0×10^{-9} M) obtained using Orange II (5.0×10^{-5} M) and eq 13 at 25 ºC and pH 8 (0.01 M phosphate).

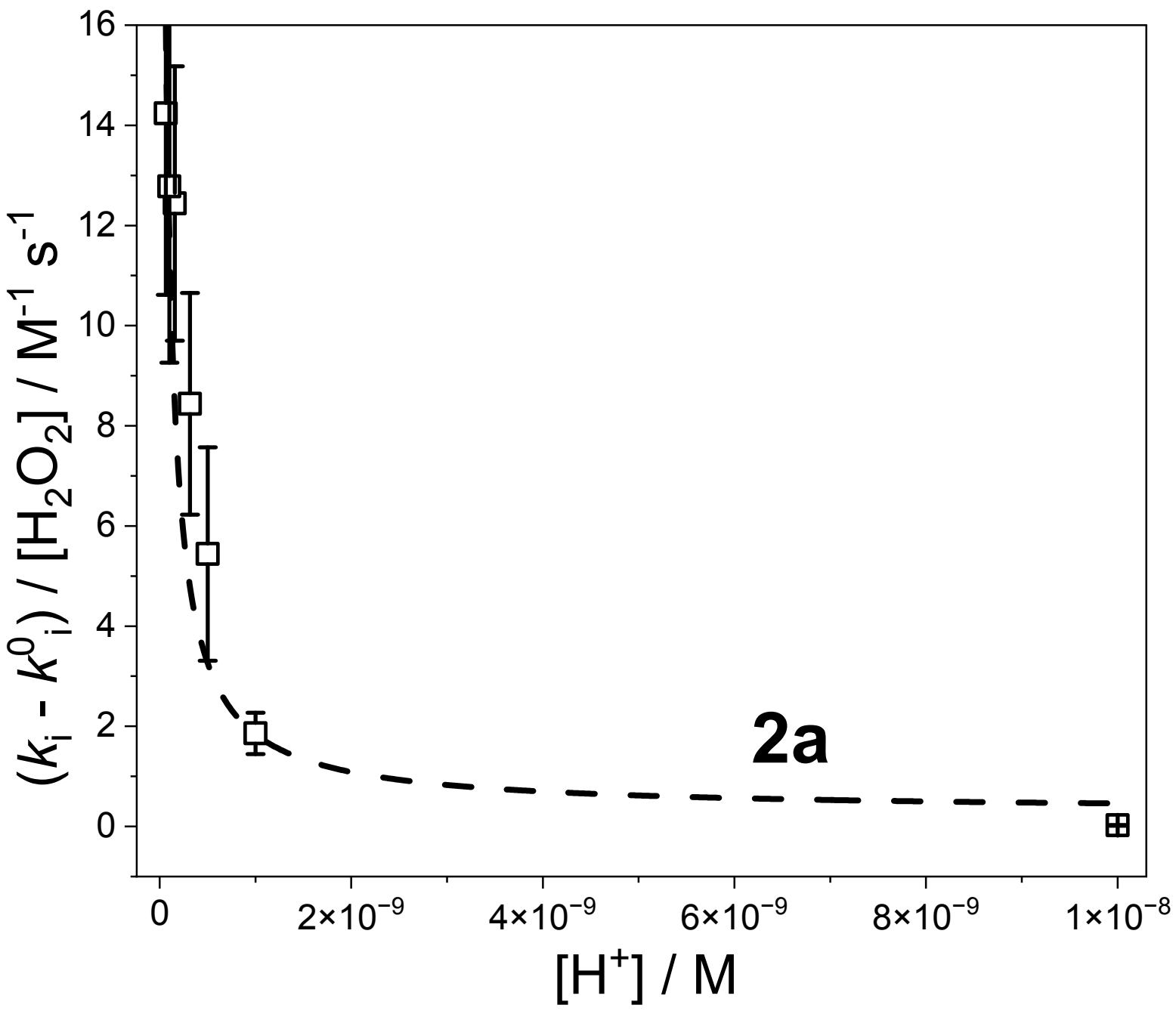

Figure S9. Fitting for **2a** k_i data points from figure 5A at pH ≥ 10.0, $[H_2O_2] = 5 \times 10^{-3}$ M according to eq 15.

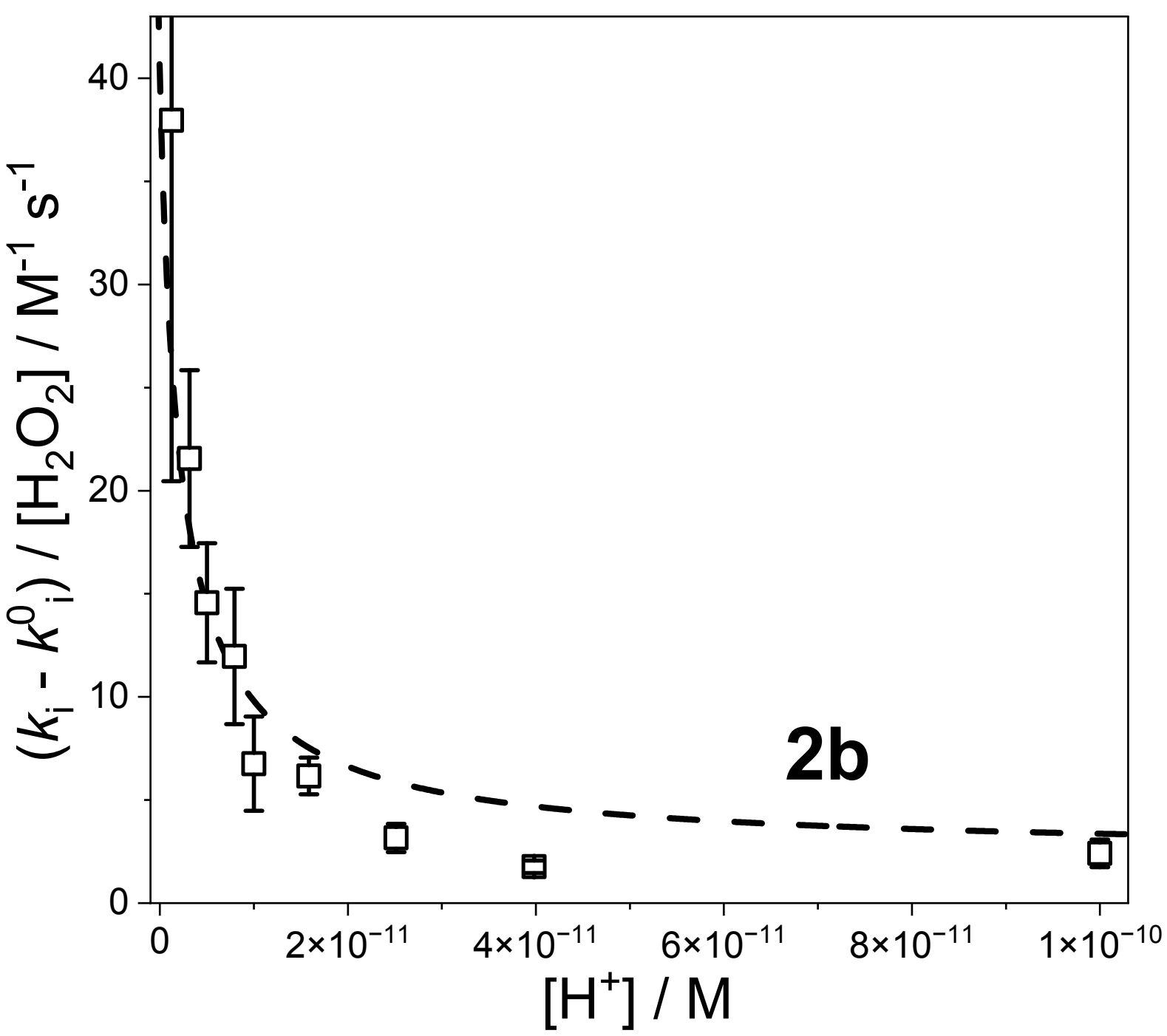

Figure S10. Fitting for **2b** k_i data points from figure 5A at pH ≥ 10.0, $[H_2O_2] = 5 \times 10^{-3}$ M according to eq 15.

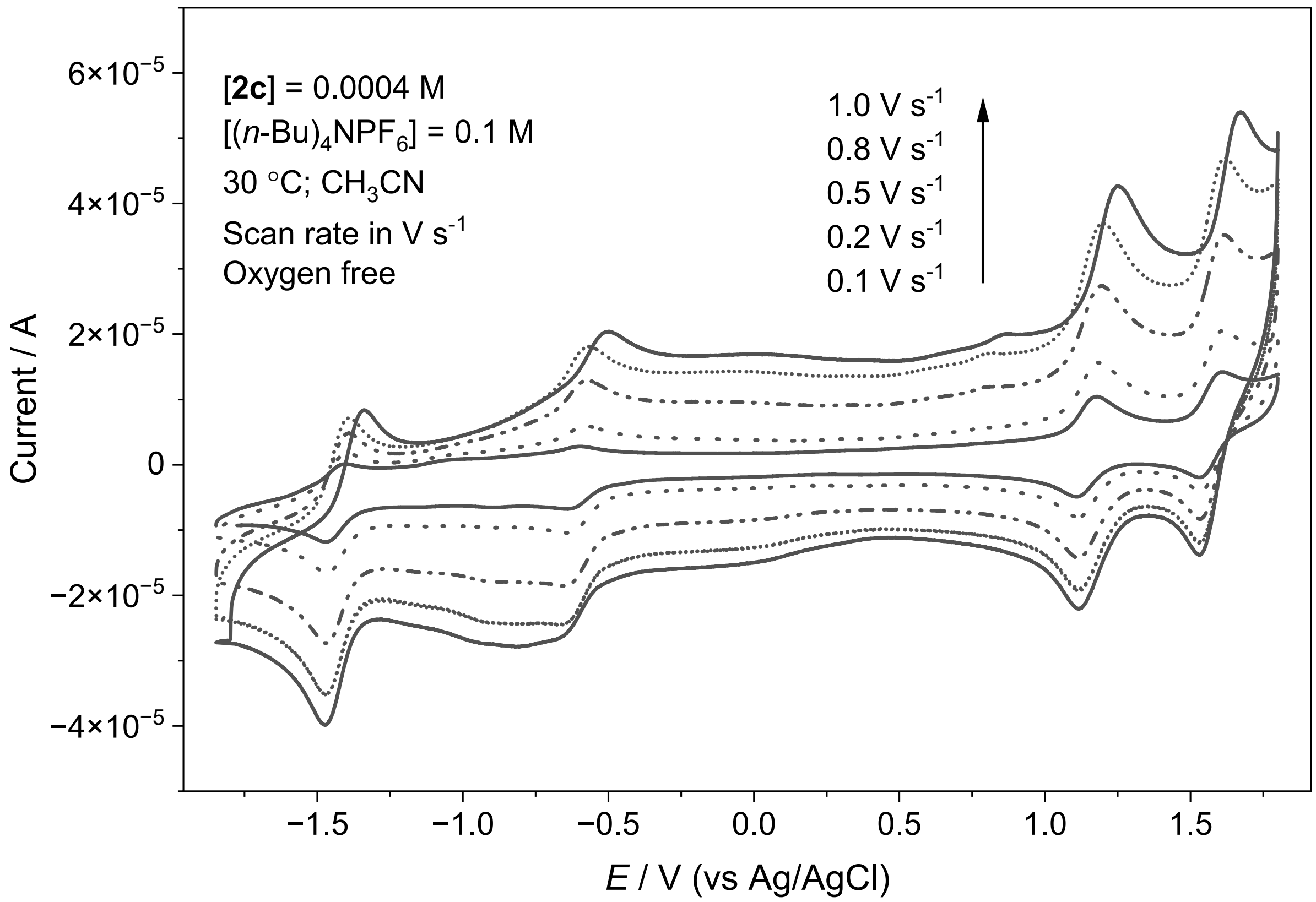

Figure S11: Cyclic voltammogram of nitrogen-deairated solution of **2c** (4.0×10^{-4} M) in MeCN containing 0.1 M (n-Bu)$_4$NPF$_6$ at increasing scan rates from 0.1 to 1.0 V s^{-1} at a glassy carbon electrode; 30 °C.

Tables

Table S1. Experimental details regarding synthesis of **2c**.

Base (equivalents)[1]	Methylating agent (equivalents)[2]	Conditions (atmosphere, solvent, temp °C)	% conversion (by ESI-MS)
KOH (10)	CH_3I (1.5)	Air, water, 25	0
Pyridine (100)	CH_3I (1000)	N_2, ACN, 40	0
Et_3N (100)	CH_3OTs (1000)	N_2, ACN, 25	1
^{t}BuOK (1.6)	$(CH_3)_3O^+$ (7)	inert, THF, 25	0 [3]
^{t}BuOK (2)	CH_3I (500)	inert, THF, 25	1
^{t}BuOK (10)	CH_3I (500)	inert, THF, 25	66
^{t}BuOK (20)	CH_3I (500)	inert, THF, 25	100 [4]
^{t}BuOK (30)	CH_3I (500)	inert, THF, 25	100

[1] **2b** was stirred with base for 1 h under Ar. Equivalence is reported with respect to **2b**.
[2] **2b** + base mixture was stirred with the methylating agent for 24 h.
[3] Demetalation of the catalyst was observed.
[4] Adopted as the optimized condition.

Table S2. Rate constants k_I and k_{II} for **2a-c** in M^{-1} s^{-1} at different pH and 25 °C. 0.01 M phosphate or carbonate was used as buffers.

NewTAML	pH	k_I	$10^{-5} \times k_{II}$
**2a**	6.0	32 ± 1	1.0 ± 0.1
	6.5	67 ± 3	2.0 ± 0.4
	7.0	260 ± 16	3.4 ± 0.7
	7.5	540 ± 20	4.3 ± 0.4
	8.0	1100 ± 60	2.3 ± 0.1
	9.0	2030 ± 100	1.4 ± 0.0
	10.0	1800 ± 100	1.3 ± 0.1
**2b**	6.0	60 ± 1	1.0 ± 0.1
	6.5	120 ± 5	4.4 ± 0.7
	7.0	340 ± 12	5.1 ± 0.5
	7.5	640 ± 20	5.9 ± 0.8
	8.0	1670 ± 70	3.8 ± 0.2
	9.0	2710 ± 95	2.1 ± 0.1
	10.0	2380 ± 210	2.1 ± 0.2
	11.0	1710 ± 70	1.5 ± 0.1
**2c**	6.0	70 ± 1	2.7 ± 0.1
	6.5	175 ± 2	6.1 ± 0.4
	7.0	410 ± 6	6.9 ± 0.5
	7.5	720 ± 30	4.2 ± 0.5
	8.0	1400 ± 60	3.0 ± 0.2

9.0	2260 ± 100	1.2 ± 0.1
10.0	2670 ± 270	1.0 ± 0.1
11.0	1340 ± 100	1.2 ± 0.1
11.5	1100 ± 90	2.8 ± 0.4
11.9	660 ± 30	3.8 ± 0.4
12.5	420 ± 10	3.0 ± 0.3

Table S3: Rate constants k_I and k_{II} obtained from the data in **Figure S4** for **2c**-catalyzed Orange II oxidation by H_2O_2 at pH 7.0 (0.01 M phosphate).

Temperature (°C)	k_I (M^{-1} s^{-1})	$10^{-6} \times k_{II}$ (M^{-1} s^{-1})
40	815 ± 20	1.0 ± 0.1
50	1280 ± 47	1.4 ± 0.2
55	1300 ± 69	1.4 ± 0.3
60	1460 ± 51	1.5 ± 0.2

Table S4. Rate constants k_i for **2c** at different pH at 25 °C in 0.01 M phosphate or carbonate buffers.

pH	$10^4 \times k_i$ / s^{-1}	Buffer used
7	51.00 ± 6.00	phosphate
8	17.20 ± 5.20	phosphate
9	7.30 ± 0.15	carbonate
10	0.60 ± 0.06	carbonate
10.5	0.74 ± 0.01	carbonate
11	0.80 ± 0.08	carbonate
11.5	2.20 ± 0.30	carbonate

Table S5. Rate constants $k_{i,0}$ and k_{i,H_2O_2} for **2a-c** and selected TAMLs **1** at pH 11 at 25 ºC in 0.01 M carbonate buffers.

Catalyst	$k_{i,0}$ / s^{-1}	k_{i,H_2O_2}/ M^{-1} s^{-1}	Reference
**2a**	[a]	[a]	This work
**2b**	0.011 ± 0.005	7.9 ± 0.9	
**2c**	(3.5 ± 0.3) ×10^{-5}	~ 0	
**1a**	0.003 ± 0.001	0.072 ± 0.007	Chanda et al.[b]
**1b**	(5.3 ± 0.8) ×10^{-4}	0.013 ± 0.001	

a) Could not be reliably measured.

b) Chanda, A.; Ryabov, A. D.; Mondal, S.; Alexandrova, L.; Ghosh, A.; Hangun-Balkir, Y.; Horwitz, C. P.; Collins, T. J. Activity-Stability Parameterization of Homogeneous Green Oxidation Catalysts. *Chemistry A European J* **2006**, *12* (36), 9336–9345. https://doi.org/10.1002/chem.200600630.

Table S6. Exact concentrations of dyes (Orange II at pH 7 – 9 or Safranine O at pH > 9), hydrogen peroxide and **2** used for collecting experimental data in Figure 5.

NewTAML	pH	[Dye] / M	[H_2O_2] / M	[**2**] / M
**2a**	7.0	5.0×10^{-5}	1.5×10^{-3}	5.0×10^{-9}
	8.0	5.0×10^{-5}	1.5×10^{-3}	1.5×10^{-8}
	9.0	5.0×10^{-5}	1.5×10^{-3}	1.5×10^{-8}
	10.0	3.0×10^{-6}	5.0×10^{-3}	5.0×10^{-8}
	10.2	3.0×10^{-6}	5.0×10^{-3}	1.0×10^{-7}
**2b**	7.0	5.0×10^{-5}	1.5×10^{-3}	5.0×10^{-9}
	8.0	5.0×10^{-5}	1.5×10^{-3}	1.5×10^{-8}
	9.0	5.0×10^{-5}	1.5×10^{-3}	1.5×10^{-8}
	10.0	3.0×10^{-6}	5.0×10^{-3}	2.5×10^{-8}
	10.4	3.0×10^{-6}	5.0×10^{-3}	2.5×10^{-8}
	10.6	3.0×10^{-6}	5.0×10^{-3}	2.5×10^{-8}
	10.8	3.0×10^{-6}	5.0×10^{-3}	2.5×10^{-8}
	11.0	3.0×10^{-6}	5.0×10^{-3}	5.0×10^{-8}
	11.1	3.0×10^{-6}	5.0×10^{-3}	5.0×10^{-8}
	11.3	3.0×10^{-6}	5.0×10^{-3}	5.0×10^{-8}
	11.5	3.0×10^{-6}	5.0×10^{-3}	5.0×10^{-8}
	11.9	3.0×10^{-6}	5.0×10^{-3}	5.0×10^{-8}
**2c**	7.0	5.0×10^{-5}	1.5×10^{-3}	5.0×10^{-9}
	8.0	5.0×10^{-5}	1.5×10^{-3}	5.0×10^{-9}
	9.0	5.0×10^{-5}	1.5×10^{-3}	5.0×10^{-9}
	10.0	3.0×10^{-6}	5.0×10^{-3}	1.0×10^{-9}
	10.5	3.0×10^{-6}	5.0×10^{-3}	1.0×10^{-9}
	11.0	3.0×10^{-6}	5.0×10^{-3}	1.0×10^{-9}
	11.5	3.0×10^{-6}	5.0×10^{-3}	1.0×10^{-9}
